# Demixer: a probabilistic generative model to delineate different strains of a microbial species in a mixed infection sample

**DOI:** 10.1093/bioinformatics/btaf139

**Published:** 2025-04-03

**Authors:** Brintha VP, Manikandan Narayanan

**Affiliations:** Department of Computer Science and Engineering, Indian Institute of Technology (IIT) Madras, Chennai, 600036, India; Center for Integrative Biology and Systems Medicine (IBSE), IIT Madras, Chennai, 600036, India; Wadhwani School of Data Science and AI, IIT Madras, Chennai, 600036, India; Department of Computer Science and Engineering, Indian Institute of Technology (IIT) Madras, Chennai, 600036, India; Center for Integrative Biology and Systems Medicine (IBSE), IIT Madras, Chennai, 600036, India; Wadhwani School of Data Science and AI, IIT Madras, Chennai, 600036, India

**Keywords:** mixed infection, latent variable modeling, mutations, strains, multi-sample analysis, parallel heuristics, Whole Genome Sequencing (WGS), CRyPTIC consortium, Tuberculosis (TB), hybrid (reference-plus-de novo) approach, Latent Dirichilet Allocation (LDA)

## Abstract

**Motivation:**

Multi-drug resistant or hetero-resistant tuberculosis (TB) hinders the successful treatment of TB. Hetero-resistant TB occurs when multiple strains of the TB-causing bacterium with varying degrees of drug susceptibility are present in an individual. Existing studies predicting the proportion and identity of strains in a mixed infection sample rely on a reference database of known strains. A main challenge then is to identify *de novo* strains not present in the reference database, while quantifying the proportion of known strains.

**Results:**

We present Demixer, a probabilistic generative model that uses a combination of reference-based and reference-free techniques to delineate mixed infection strains in whole genome sequencing (WGS) data. Demixer extends a topic model widely used in text mining to represent known mutations and discover novel ones. Parallelization and other heuristics enabled Demixer to process large datasets like CRyPTIC (Comprehensive Resistance Prediction for Tuberculosis: an International Consortium). In both synthetic and experimental benchmark datasets, our proposed method precisely detected the identity (e.g. 91.67% accuracy on the experimental *in vitro* dataset) as well as the proportions of the mixed strains. In real-world applications, Demixer revealed novel high confidence mixed infections (101 out of 1963 Malawi samples analysed), and new insights into the global frequency of mixed infection (2% at the most stringent threshold in the CRyPTIC dataset) and its significant association to drug resistance. Our approach is generalizable and hence applicable to any bacterial and viral WGS data.

**Availability and implementation:**

All code relevant to Demixer is available at https://github.com/BIRDSgroup/Demixer.

## 1 Introduction

Tuberculosis (TB) is a contagious disease caused by the bacterium *Mycobacterium tuberculosis (M. tb)*, and is treated using a combination of antibiotic drugs taken over a prolonged period. TB has been around for thousands of years, and treating it has become increasingly difficult in recent times due to the evolving nature of *M. tb*. Specifically, the growing resistance of different strains of *M. tb* (referred to simply as strains of TB or TB strains hereafter) to commonly used drugs (Seung *et al.* 2015, Shanmugam *et al.* 2022) extends the treatment regimen from months to years (Mase and Chorba 2019). TB infections caused by a single drug-sensitive strain are highly susceptible to first-line treatments, including isoniazid and rifampicin (Seung *et al.* 2015). In contrast, mixed infections involving multiple strains of TB can complicate treatment and are more challenging to manage (Behr 2004, Huang *et al.* 2010). Mixed TB infection in an individual can arise due to transmission of different strains to the individual (co-infection), evolution of an existing strain during treatment (McIvor *et al.* 2017), or when immunity is compromised due to poor treatment conditions or HIV-TB co-infection (Cohen *et al.* 2012).

Determining the identity (mutational profile) and proportion of individual strains in a mixed infection TB sample could greatly aid clinical decisions during treatment. Mixture of bacterial strains in a sample can be identified using two broad approaches: (i) traditional molecular subtyping based approaches like Mycobacterial Interspersed Repetitive Unit–Variable Number Tandem Repeat (MIRU-VNTR), which are relatively cost-effective but have limitations (e.g. MIRU-VNTR failed to detect drug-resistant strains in certain mixed infection cases (Hingley-Wilson *et al.* 2013)); and (ii) modern whole genome sequencing (WGS) based methods, like ones enabled by Illumina/Nanopore technologies, which can generate high-resolution data that if properly analysed can identify and quantify different strains in a sample with high sensitivity (Iketleng *et al.* 2018).

The WGS-based methods for quantifying strains can be classified into two main categories: reference-based and reference-free. TBProfiler (Phelan *et al.* 2019), a popular reference-based tool uses a comprehensive database of reference lineages and sublineages to delineate the strains in a sample. QuantTB is a state-of-the-art reference-based method that uses a curated list of single nucleotide polymorphisms (SNPs) from select TB genome sequences to deconvolve mixed infection (Anyansi *et al.* 2020a). QuantTB’s reliance on the diversity of the reference database limits its ability to detect new strains in the absence of related strains in the database. MixInfect (Sobkowiak *et al.* 2018), a reference-free approach uses the ratio of heterozygous alleles to quantify the mixed infection proportions. As a result, this method reveals only the strains’ proportions but not their identity (mutational profile). SplitStrains (Gabbasov *et al.* 2021) is a new statistical-based method that aligns with MixInfect due to its non-reliance on the reference database and uses a likelihood ratio test to resolve the heterogeneity of the samples. But, SplitStrains like MixInfect cannot reveal the identity of the strains.

A major challenge therefore with existing WGS-based methods like QuantTB is their strict reliance on reference databases, and with reference-free approaches MixInfect or SplitStrains is their difficulty to infer the mutational profile of *de novo* strains. To address these challenges, we introduce a hybrid (reference-plus-*de novo*) approach called Demixer to infer the individual strain proportions and their corresponding mutations present in a WGS dataset. Demixer uses a probabilistic generative model assuming that each sample’s WGS reads are generated via a process that first generates the proportions of different strains mixed in a sample (Sankaran and Holmes 2019), and then the reads corresponding to the mixed strains. By learning the parameters of this generative process, including those corresponding to the latent variable (hidden or unknown) of each WGS read, we can accurately infer/predict the distribution of strains in a new sample. The Demixer model extends upon the Latent Dirichlet Allocation (LDA) framework (Blei *et al.* 2003), a popular topic model in text mining, due to its simplicity and interpretability compared to word-embedding-based models (Asudani *et al.* 2023).

To better suit the unique characteristics of genomic data, we introduced specific modifications to the LDA model, referred to as SNP-LDA. The SNP-LDA model, along with preprocessing and postprocessing steps, collectively constitute our method, Demixer.

Demixer’s multi-sample analysis capability allows it to refine the mutational profile of a reference strain (for instance to augment known mutations of the strain in the reference database with additional mutations present in the samples), extract the mutational profile of a *de novo* strain present in multiple samples, and quantify their proportions by incorporating the commonalities and differences among multiple samples. To improve Demixer’s speed and scalability, we have implemented parallelization heuristics. Demixer was effective in delineating mixed strains in various synthetic and real-world datasets (see Supplementary Table S1 for dataset details). For instance, Demixer inferred mixed strains’ identities in an *in vitro* dataset with an accuracy (91.67%) comparable to or better than existing methods. Demixer-estimated strain proportions even at 20× coverage with 99% accuracy in synthetic benchmarks generated at different coverage levels, and proportions of *de novo* strains which were absent in the reference database (with mean relative error 0.061) in another synthetic dataset. On application to real-world Malawi dataset, Demixer detected 101 new (previously unreported) mixed infections with high confidence out of the 1963 samples analysed; and when applied to CRyPTIC dataset, Demixer revealed new insights into the global prevalence of mixed infection and its association with drug resistance. Furthermore, the parameters of Demixer obtained by training on CRyPTIC dataset can be used to test new samples, making it more suitable for clinical diagnosis.

## 2 Materials and methods

### 2.1 Description of Demixer method

**Figure 1.**
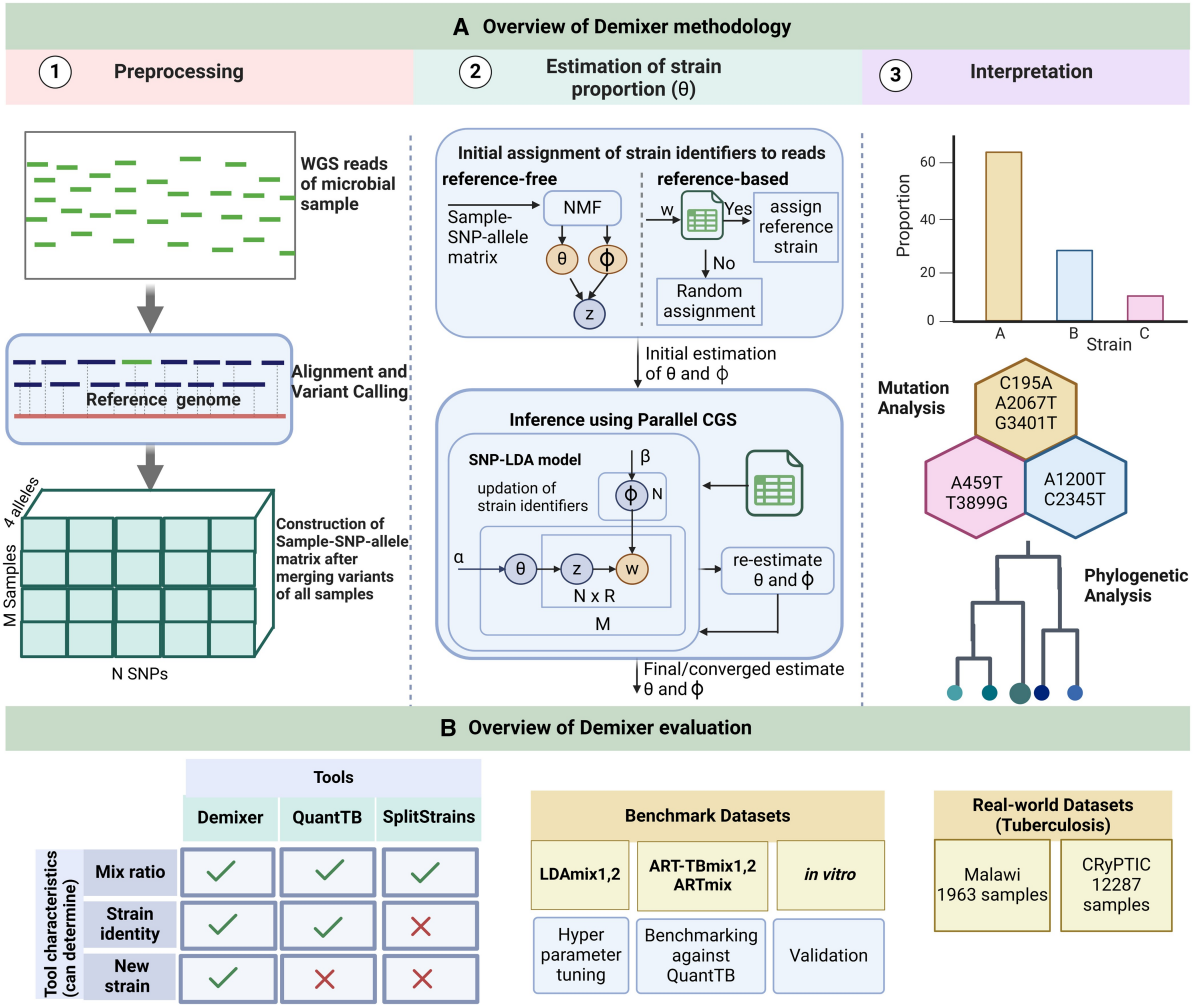
Our Demixer overview. (A) Illustrated here are the three steps of our Demixer method (preprocessing of WGS reads to generate the M×N×4 Sample-SNP-allele matrix, SNP-LDA model based inference of the Sample-Strain proportions θ from this matrix, and interpretation of the learnt mutations). The matrix of parameters of our SNP-LDA model, θ and ϕ (Strain-SNP-allele distributions), are estimated using the parallelized Collapsed Gibbs Sampling (CGS) inference algorithm (refer Supplementary Figs S1 and S2). (B) Different synthetic/real-world datasets and methods used to assess the effectiveness of Demixer in estimating the strain identities and their proportions.

Demixer uses the WGS reads of each sample and a database of mutations in reference strains as inputs to determine the composition of the reference and *de novo* strains present in the samples. Figure 1A illustrates the three steps of Demixer (as described below) and Fig. 1B shows the different datasets and state-of-the-art methods used to assess the performance of Demixer under diverse settings.

#### 2.1.1 Preprocessing

Preprocessing the WGS reads of the input microbial samples involves performing read alignment and variant calling to generate the variants file (in .vcf format) for each sample, and merging them across all M samples to generate the Sample-SNP-allele matrix S. Details of the preprocessing pipeline are in Supplementary Section S1.3.1.

#### 2.1.2 Estimation of strain proportion

Demixer learns the strain proportion of samples via the SNP-LDA model. The notations, description, inference algorithm and heuristics adopted to improve the SNP-LDA model are explained in this section.

*SNP-LDA model notations*: The notations used to describe SNP-LDA model are given below. To simplify exposition, vectors, (two-dimensional) matrices, and three- or higher-dimensional tensors are all referred to as matrices in the text (with the matrix dimensions or sizes indicated as subscripts of the form $dim_{1}\times dim_{2}\times \cdots$). We will need the indicator function $1_{\text{condition}}$, which is defined as 1 if the condition (logical expression) evaluates to true, and 0 otherwise.

- Inputs:
  -
M is the number of samples, N is the number of SNPs (obtained after preprocessing), and each read of a SNP supports one of the SNP’s four alleles: A, C, G, or T (also denoted 1, 2, 3, 4, respectively, when used as index of a matrix).
  -
S or $S_{M\times N\times 4}$ – Sample-SNP-allele matrix of size M×N×4. Here, $S_{m,n,v}$ is the number of reads supporting allele v at nth SNP in the mth sample.
  -
R – number of reads mapping to each SNP. This assumption of same number of reads per SNP simplifies exposition (however our model/implementation can easily handle different number of reads mapping to each SNP).
  -
$w_{m,n,r}$ – allele supported by the rth read mapping to SNP n in sample m. Note that S can be constructed from all $w_{m,n,r}$ observations as $S_{m,n,v}=\sum_{r=1}^{R}1_{\left(w_{m,n,r}==v\right)}$.
  -
K – number of possible strains.
- Model parameters:
  - Sample-Strain distribution, $\theta_{M\times K}$.
  - Strain-SNP-allele distribution, $\phi_{K\times N\times 4}$
- Hyperparameters: α and β, which are the parameters of the Dirichlet distributions θ and ϕ, respectively.
-
$z_{m,n,r}$ is the latent variable indicating the strain assigned to read r of *n*th SNP in sample m.
-
$C_{k,m,n,v}$ represents the number of reads of sample m at *n*th SNP that supports allele v and is assigned to strain k (i.e. reads r for which $w_{m,n,r}=v$ and $z_{m,n,r}=k$).
- Whenever an index is replaced by * in any variable’s notation above, we sum the variable over all possible values of the index. For instance, $C_{*,m,n,v}=\sum_{k=1}^{K}C_{k,m,n,v}$, $S_{m,n,v}=C_{*,m,n,v}$, and $S_{m,n,*}=R$.

*SNP-LDA model description*: Each sample m is assumed to contain a mixture of K strains and is composed of a set of N SNPs, each quantified by R reads that reveal the composition of alleles at the SNP. The random variables of the model include the parameters $\theta_{m}$ (distribution of strains in mth sample) and $\phi_{k,n}$ (distribution of the four alleles at SNP n for strain k), the latent variable $z_{m,n,r}$ (strain assigned to the read denoted (m,n,r)), and the observed allele $w_{m,n,r}\in \{A,C,G,T\}$. The generative process of our SNP-LDA model follows:

- For each WGS sample m, generate $\theta_{m}$  ∼ Dirichlet(α).
- For each strain k and SNP n, generate $\phi_{k,n}$  ∼ Dirichlet$\left(\beta_{n}\right)$.
- To generate rth read $w_{m,n,r}$ at each SNP n in each sample m:
  - Generate $z_{m,n,r}∼$ Multinomial$\left(\theta_{m}\right)$.
  - Generate $w_{m,n,r}∼$ Multinomial$\left(\phi_{z_{m,n,r},n}\right)$.

‘Generate’ here refers to sampling a value for a random variable (r.v) from the specified distribution. The model parameters are learnt from the observed data w using a Gibb’s sampling based inference algorithm explained below.

Note that most applications of LDA in bioinformatics use published LDA models directly to address specific challenges (Liu *et al.* 2016). We had to modify and extend the LDA model to capture the reads assigned to the four alleles of a SNP (which has different semantics than words in a text); and we also propose a parallelization heuristic to speedup the CGS inference algorithm on our Demixer SNP-LDA model (Fig. 1A). Compared to traditional LDA model’s generative process reviewed in Supplementary Section S1.1, SNP-LDA model coherently models all the four alleles at each SNP position by using a ϕ matrix of size K×N×4. The advantage of this coherent modeling in SNP-LDA compared to LDA is also demonstrated empirically (see Section 3 and Supplementary Section S1.6.1).

*SNP-LDA model likelihood*: The generative process of the SNP-LDA model (also captured in the DAG or Directed Acyclic Graph structure in Fig. 1A.2) can be used to derive the likelihood of the model as shown below. First, let $w_{m,\cdot ,\cdot }$ refer to the set of alleles observed in all R reads of N SNPs of sample m, and let $z_{m,\cdot ,\cdot }$ be similarly defined as the set of the corresponding latent variables. Then, the joint distribution is given by:

$$\begin{array}{c} P(w,z,\theta ,\phi |\alpha ,\beta )=P(\phi |\beta )P(w,z,\theta |\alpha ,\phi )\\ =P(\phi |\beta )\prod_{m=1}^{M}P(w_{m,\cdot ,\cdot },z_{m,\cdot ,\cdot },\theta_{m}|\alpha ,\phi )\\ =P(\phi |\beta )\prod_{m=1}^{M}P(\theta_{m}|\alpha )P(z_{m,\cdot ,\cdot }|\theta_{m})P(w_{m,\cdot ,\cdot }|z_{m,\cdot ,\cdot },\phi )\\ =\left(\prod_{k=1}^{K}\prod_{n=1}^{N}P(\phi_{k,n}|\beta_{n})\right)\left(\prod_{m=1}^{M}P(\theta_{m}|\alpha )\right)\\ \prod_{m=1}^{M}\prod_{n=1}^{N}\prod_{r=1}^{R}P(z_{m,n,r}|\theta_{m})P(w_{m,n,r}|\phi_{z_{m,n,r},n}) \end{array}$$

Here, P refers to the probability mass function for a discrete r.v and probability density function for a continuous r.v.

*SNP-LDA inference algorithm (parameter estimation)*: The posterior distribution of the latent variables given the observed data P(θ,ϕ,z | w,α,β) is generally intractable, and Gibbs sampling offers one way to perform inference and parameter estimation by sampling from this posterior. The collapsed variant of a Gibbs Sampler (CGS) returns samples from the posterior P(z | w,α,β), where the multinomial parameters θ and ϕ have been marginalized (collapsed) out. Once the originating strain of each read given by $z=\{z_{m,n,r}\}$ is known (i.e. assigned via CGS samples), θ and ϕ can be easily estimated (see Supplementary Section S1.3.2).

One update step of CGS considers a specific read denoted (m,n,r), and assigns it to a strain (i.e. $z_{m,n,r}$ is sampled/updated) given the strain assignment of all other reads (denoted by $z_{-(m,n,r)}$). Let $w_{m,n,r}=v\in \{A,C,G,T\}$ and $w_{-(m,n,r)}$ denote the alleles at all other reads. If $z_{m,n,r}=k^{\prime }$ before the updation step, define $C^{-(m,n,r)}$ to be a copy of C but with one entry $C_{k^{\prime },m,n,v}^{-(m,n,r)}$ decremented by 1 (in order to remove the count contribution of the current read). Then, the conditional required for a CGS update step obtained from marginalizing the joint distribution above is given by Equations (1) and (2):

(1) $$\begin{array}{c} P(z_{m,n,r}=k\;|\;z_{-(m,n,r)},w,\alpha ,\beta )\\ \;\;\;\;\propto P(z_{m,n,r}=k,z_{-(m,n,r)},w\;|\;\alpha ,\beta ) \end{array}$$

 

(2) $$\begin{array}{c} =\int \int P(z_{m,n,r}=k,z_{-(m,n,r)},w,\theta ,\phi \;|\;\alpha ,\beta )d\theta d\phi \\ \;\;\;\;\propto (C_{k,m,*,*}^{-(m,n,r)}+\alpha_{k})\times \frac{\left(C_{k,*,n,v}^{-(m,n,r)}+\beta_{n,v}\right)}{\left(C_{k,*,n,*}^{-(m,n,r)}+\beta_{n,*}\right)} \end{array}$$

 

$$\begin{array}{c} \;\;\;\;(\text{recall}\;w_{m,n,r}=v;\;\;\text{see}\;\text{Suppl}\;\text{Section}\;1.3.2\;\text{for}\;\text{derivation}) \end{array}$$

During initialization, i.e. before the Gibbs sampling iterations, each read is assigned to a random strain (which is picked uniformly at random from all the K strains; in other words, the strains of reads are initialized randomly). Then during one iteration/epoch of CGS, the strain assignment of all reads across all SNPs and samples are updated one at a time sequentially as per Equation (2). These CGS iterations are repeated 1000 times (as it leads to convergence in most of our experiments).

*Heuristics to improve SNP-LDA model*: We propose the following heuristics to improve strain identification and reduce the running time of the SNP-LDA model (see Supplementary Section S1.3.2 for details of different heuristics).

- *Initialization heuristic*: We explored two different ways to initialize the strains of WGS reads: using NMF, or using the information of known strains and their related mutations to initialize the reads arising from those mutations (see Supplementary Section S1.2). The four variants based on these initialization heuristics (as well their combinations) can be categorized into two reference-free and two reference-based (also known as hybrid) variants as follows: (i) SNP-LDA—a vanilla variant that initializes the strain of each read randomly; (ii) NMF SNP-LDA [also denoted (NMF, SNP-LDA)]—variant that initializes the strains of reads in the SNP-LDA model using the NMF outputs; (iii) (non-NMF) Hybrid SNP-LDA—a reference-based variant that initializes the strain of reads containing reference mutations/SNP-alleles to the corresponding reference strain and other reads randomly; and (iv) (NMF, hybrid SNP-LDA)—another reference-based variant that initializes the strain of reads containing mutations of a user-revealed reference strain p to p itself and other reads using the NMF outputs (this variant has only limited application in synthetic benchmarks where a reference strain in a sample is revealed, and the benchmark task is to find the remaining *de novo* strains in the sample and all strain proportions). Across the SNP-LDA model variants above, we employed five different hyperparameter combinations (1,0.01), (0.01,A), (0.01,B), (A,0.01), and (B,0.01). The first element in each set corresponds to the α hyperparameter and the second element to the β hyperparameter (see Supplementary Section S1.6.2 for details of A and B). We used synthetic datasets LDAmix1 and LDAmix2 (see Section 2.3 and Supplementary Section S1.4 for the description of datasets) to select the best model variant and hyperparameter configuration. We also considered a baseline approach, NMF-only, where NMF outputs are the final estimates of ϕ and θ.
- *Heuristic for choosing* K: The number of strains K is set to $K^{\prime }+2$, where $K^{\prime }$ correspond to the number of reference strains whose unique mutations (SNP-alleles) are present in the input samples and is incremented by 2 to allow the identification of *de novo* strains. The intuition behind the choice of K is discussed in detail in Supplementary Section S1.3.2.
- *Weight heuristic*: As the number of reference SNP-alleles could be quite low compared to the total number of SNP-alleles in a large-scale setting, we give higher weightage for the reference SNP-alleles and the corresponding CGS update incorporating the weights is given in Supplementary Equation (6) (see Supplementary Section S1.3.2 and Tables S2 and S3).
- *Parallelization heuristic*: The samples are processed in parallel to enable the speedup of CGS algorithm as depicted in Supplementary Fig. S2. Such parallelization is enabled by adopting a delayed update strategy (see Supplementary Section S1.3.2).

The different steps of SNP-LDA’s parameter estimation involving the different heuristics are also outlined in Algorithm 1 in Supplementary Information (also see Supplementary Section S1.6.4 for implementation details).

We also performed an ablation study to evaluate the performance of each heuristic by excluding either the K or weight heuristic, with or without the reference strains on LDAmix1 and *in vitro* datasets. We considered six heuristic combinations by excluding or including one of the three heuristics: initialization using reference strains (indicated by R), K and weight (indicated by W). For simplicity, we denote the combination of these heuristics as a trio (R or $\bar{R}$, K or $\bar{K}$, W or $\bar{W}$), where the bars over the letters indicate that the heuristic is excluded. The six combinations tested are: ($\bar{R},\bar{K},\bar{W}$), ($\bar{R}$, K, $\bar{W}$), (R, $\bar{K}$, $\bar{W}$), (R, $\bar{K}$, W), (R, K, $\bar{W}$), and (R, K, W). Here (R, K, W) correspond to the default Demixer model, which includes all the heuristics. The configurations ($\bar{R}$, K, W) and ($\bar{R}$, $\bar{K}$, W) are not considered, as weights can be added only to the reference mutations. Additionally, the speedup resulting from parallelization heuristic was also assessed using different number of processor cores on *in vitro* and ART-TBmix2 (90×–10×) datasets.

#### 2.1.3 Postprocessing/interpretation steps

To enhance the interpretability of our method’s predictions, we perform the following postprocessing steps on the strains inferred by Demixer:

- Mapping inferred to reference strains.
- Fine-tuning the mapped strains using the lineage tree.
- Quality checks using SNP plots/modes ($o_{m}$ and $f_{m}$ values) and *de novo* filtering.
- Hierarchical clustering of the inferred strains, and *in vitro* dataset processing.

Please refer Supplementary Section S1.3.3 for a detailed description of each postprocessing step, and Algorithm 2 in Supplementary Information for the order in which these steps are done. Supplementary Section S1.3.3 also provides the definition of the $f_{m}$ value—briefly, $f_{m}$ value of the mth sample, expressed in terms of the number of SNPs, is used to decide if a mixed infection call is of low, medium or high confidence. This quality assessment is based on the distribution of minor allele proportions of the heterozygous SNPs in the sample.

### 2.2 Evaluation metrics

We use the metrics such as relative error (RE) and F1 score to compare the performance of Demixer with other models and state-of-the-art methods on different benchmark datasets (see Supplementary Section S1.5 for details).

### 2.3 Datasets used in the analysis

We used the following synthetic and real-world datasets in our study to tune and evaluate Demixer (see Supplementary Section S1.4 for details and Supplementary Table S1 for dataset sizes).

*LDAmix1 and LDAmix2 datasets*: These synthetic datasets are created using our SNP-LDA model’s generative process to evaluate Demixer’s potential in delineating the mixed strains under different hyperparameter combinations. Both LDAmix1 and LDAmix2 consist of 100 samples each, with each sample in LDAmix1 containing 3 strains and each sample in LDAmix2 containing four strains.

*ART-TBmix1 and ART-TBmix2 datasets*: To mimic the benchmark dataset used to evaluate QuantTB, we generated two standard datasets (ART-TBmix1 and ART-TBmix2) as per the simulation procedure described in Gabbasov *et al.* (2021), Anyansi *et al.* (2020a) using ART simulator (Huang *et al.* 2012). Both ART-TBmix1 and ART-TBmix2 consists of 800 samples each, at four distinct levels of coverage: 10×, 20×, 90×–10×, and 70×–30×, each with 200 samples. A sample in 10× and 20× datasets is mixed with four strains, whereas two strains are mixed in 90×–10× and 70×–30× datasets using the corresponding coverage levels.

*ARTmix dataset*: ARTmix was generated to evaluate the performance of Demixer in detecting *de novo* strains, when information about the other strains are revealed in the reference database. Each of the 50 samples in this dataset comprises either 1, 2, or 3 strains, which are selected from a set of seven strains obtained by replacing 100 SNPs in the reference H37Rv genome.

*In vitro dataset*: An *in vitro* dataset of 48 samples obtained by experimentally mixing the DNA of two *M. tb* samples (belonging to lineages 1–4) in one of these proportions: 0.7/0.3, 0.90/0.10, 0.95/0.05, and 1.00/0.00. This realistic benchmark dataset from an earlier study (Sobkowiak *et al.* 2018) is used to compare Demixer with other published methods.

*Malawi and CRyPTIC datasets*: Two real-world datasets, comprising 1963 TB isolates obtained from patients in Malawi (Sobkowiak *et al.* 2018) and 12 287 TB isolates obtained across 23 countries by CRyPTIC (CRyPTIC Consortium, 2022a) has been used to detect mixed infections. The CRyPTIC dataset is also examined to assess the impact of mixed infection on drug resistance.

## 3 Results

### 3.1 Model selection and hyperparameter tuning using synthetic datasets

**Figure 2.**
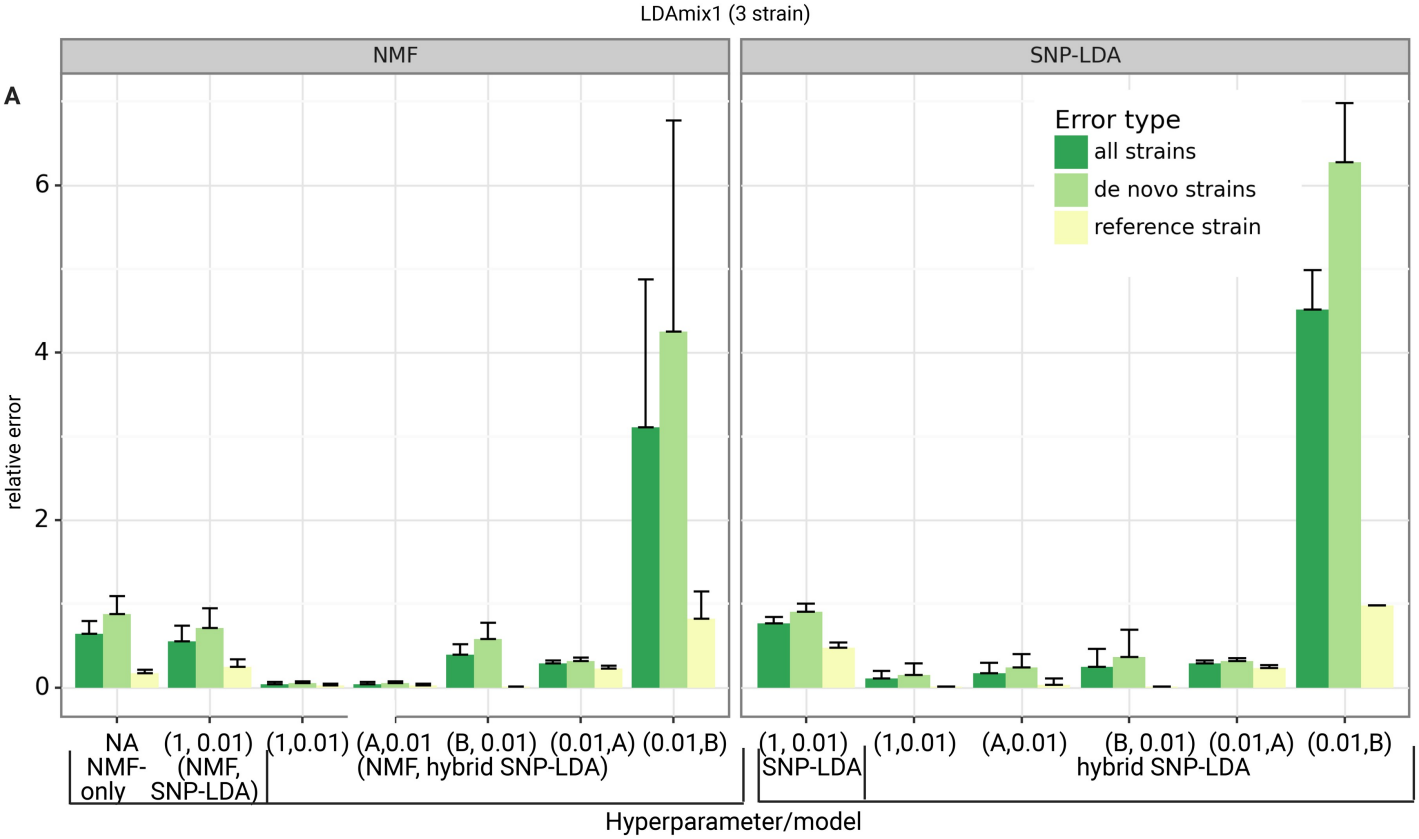
Performance of different SNP-LDA model variants on LDAmix1 dataset. Model variants are indicated in a pairwise tuple format (see Supplementary Section S1.3.2). Each dataset has been generated 10 times (i.e. in 10 runs using different random seeds). The *x*-axis indicates the different methods tested (with their hyperparameter/model combinations; see Supplementary Section S1.6.2), and the *y*-axis indicates the relative error between the actual and predicted strain proportions averaged across the 10 runs (with error bar overall length being the standard deviation across the 10 runs). The error type indicates the error in determining all the strains, solely reference strains, and only the *de novo* strains in the samples within each dataset.

The selection of appropriate hyperparameters for a probabilistic model such as LDA can play a role in improving its performance in text mining tasks (Wallach *et al.* 2009). Since our SNP-LDA is a newly proposed LDA model, we wanted to first check if the model is sensitive or robust to the choice of hyperparameters and other settings. We compared the performance of different variants of SNP-LDA model with different hyperparameter settings on two synthetic datasets, LDAmix1 and LDAmix2 (see Fig. 2 and Supplementary Fig. S3).

Using relative error as the evaluation measure, we observed that all model variants, except the one with hyperparameter (0.01,B) (see Section 2), were able to accurately predict the reference strain proportions. For predicting *de novo* strain proportions, we found the error to be lower for most hybrid SNP-LDA combinations, indicating the effectiveness of using information (mutations) of known strains on top of the SNP-LDA model. Also, the symmetric hyperparameter setting (1,0.01) was similar or better in performance compared to the other asymmetric hyperparameter settings; so we will use the former for all future analyses.

The (NMF, hybrid SNP-LDA) combined models leaving out the one with hyperparameter (0.01, B) have lower relative error than NMF-only models (indicating the benefit from probabilistic treatment of uncertainty in SNP-LDA) and non-NMF models as well. But, (NMF, hybrid SNP-LDA) models have limited applicability beyond synthetic benchmarks as discussed in Methods; further it can be challenging to assign reference SNP-allele labels to NMF-inferred strains from real-world data. Therefore, we use the (non-NMF) hybrid SNP-LDA model with hyperparameter (1,0.01) as the default model for Demixer for all subsequent analyses. We have also compared our Demixer to the traditional LDA model on a 2-strain dataset when no reference strains are known (see Supplementary Section S1.6.1), and the better performance of our model on this dataset (Supplementary Fig. S4) supports why a new model such as ours is needed for estimating strain proportions in WGS samples.

The ablation analysis demonstrated that incorporating prior information is crucial for accurately identifying strain proportions, regardless of the value of K. For instance, the average relative error reduced from 1.9 (without prior) to 0.29 (with prior) in the *in vitro* dataset. However, selecting the optimal value of K is essential in the absence of reference strains and the effectiveness of K and weight heuristics largely depends on the dataset (see Supplementary Fig. S5 and Table S6). Furthermore, the parallelization heuristic led to a substantial speedup in runtime, with nearly 7×–10× improvement compared to the non-parallelized setting. This speedup is evident in both small and medium-sized datasets tested (see Supplementary Fig. S6).

### 3.2 Demixer performs better than QuantTB in the *in vitro* dataset

**Figure 3.**
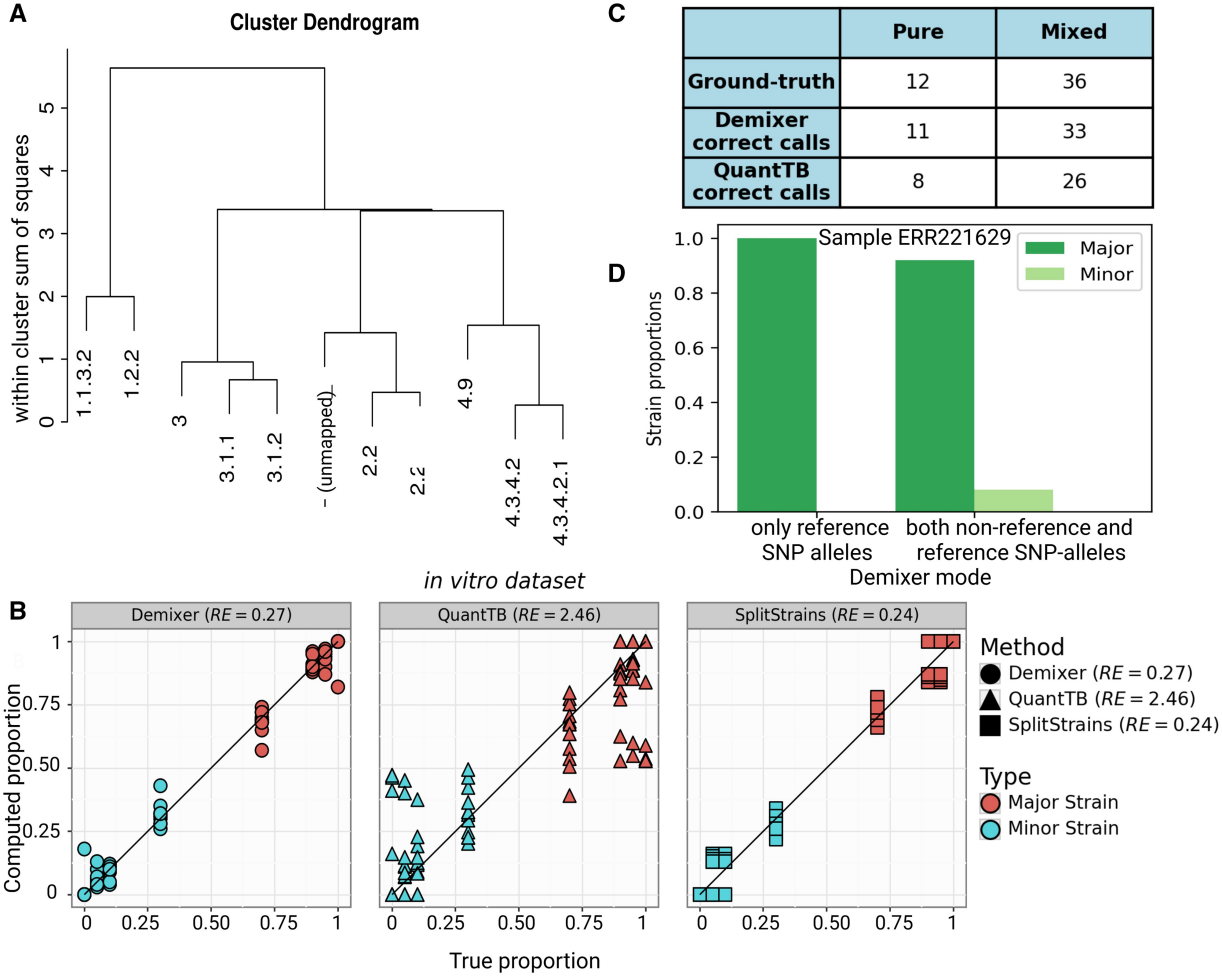
Demixer versus other methods on *in vitro* dataset. (A) The hierarchical relationship between the strains inferred by Demixer in the *in vitro* dataset. (B) Compares the predicted versus actual proportions of the major/minor strain in each sample. (C) The number of samples for which the strains inferred by Demixer (or QuantTB) mapped correctly to the lineages present in the sample. (D) The strain proportions identified by Demixer for the sample ERR221629, which is actually a mixed sample with ground-truth strain proportions 0.90/0.10. SplitStrains can detect only proportions and not strain identities, so it is not shown in panel C.

We compared the performance of our Demixer with state-of-the-art methods QuantTB and SplitStrains using the *in vitro* dataset. In this work, QuantTB and SplitStrains are run on the samples using their corresponding default settings and parameter values. We use the hierarchical clustering tree of the strains inferred by Demixer (Fig. 3A) to group the strains into two clusters (see Supplementary Section S1.3.3), and thereby estimate the major and minor strain proportions. These predicted proportions by Demixer are very similar to the actual proportions and the associated relative error (RE) is much lower than QuantTB and comparable to SplitStrains (Fig. 3B). Demixer was also better than QuantTB in inferring the lineages of strains correctly (Fig. 3C; see also Supplementary File D1 for the inferred lineages). Demixer inferred strains related to lineages 1–4 correctly in most of the samples, except for one unmapped strain in a sample (i.e. closer to lineage 2 in the hierarchical tree, and explained in detail in Supplementary File D1).

Demixer uses both reference and non-reference SNP-alleles to perform its inference, and this offers an advantage as demonstrated by Demixer correctly identifying the sample ERR221629 (with ground-truth proportion 0.90/0.10) as mixed; because when only reference SNP-alleles were used, Demixer incorrectly classified this sample as pure (Fig. 3D).

### 3.3 Evaluating Demixer on QuantTB and novel-strain-identification benchmarks

**Figure 4.**
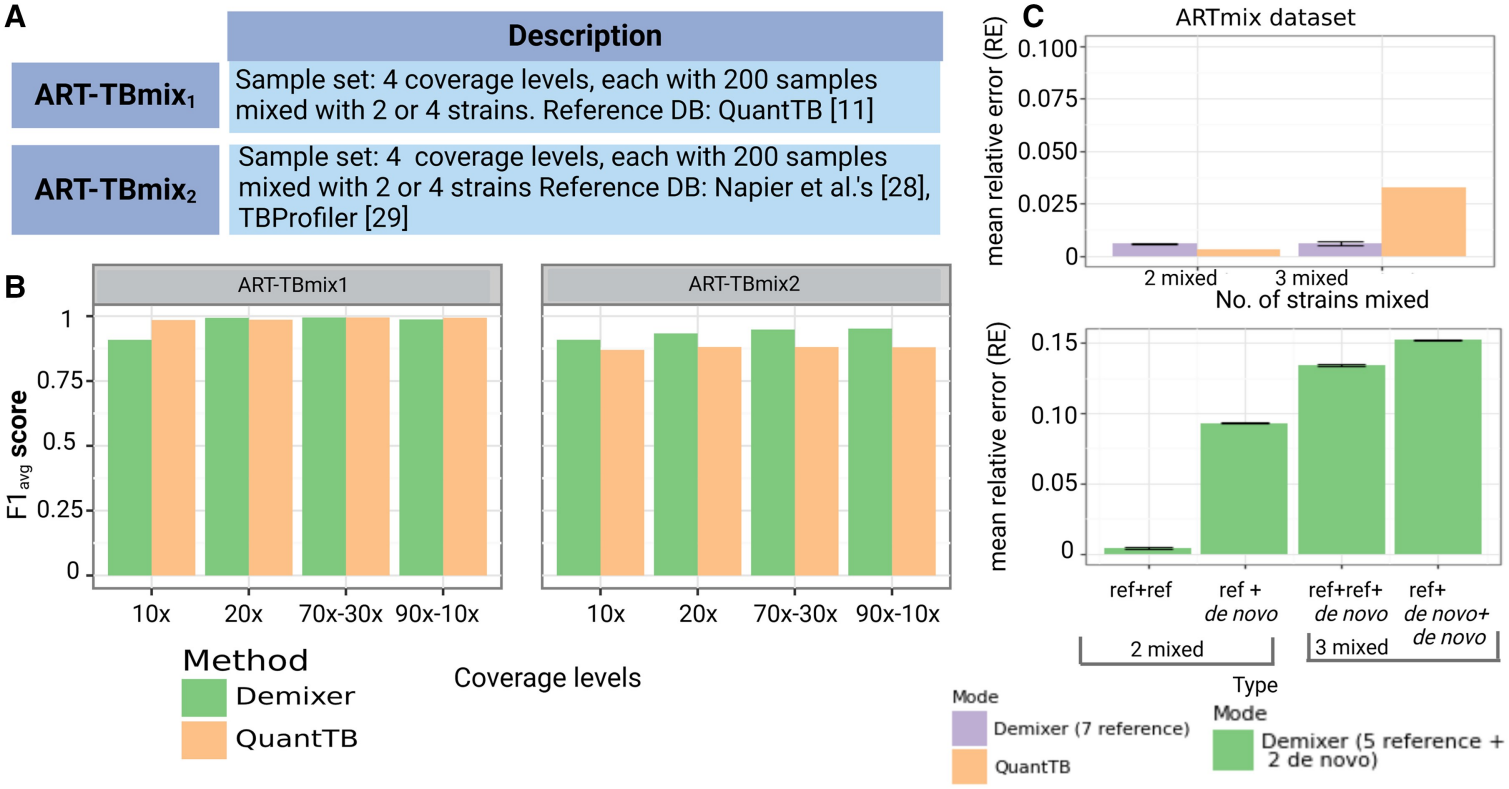
Comparison of Demixer versus QuantTB. (A) The table highlights the number of samples present in the datasets ART-TBmix1 and ART-TBmix2. The strains of ART-TBmix1 and ART-TBmix2 are generated using the SNP-alleles of strains based on QuantTB (Anyansi *et al.* 2020a) and (Napier *et al.* 2020) works, respectively. (B) $F1_{\mathrm{avg}}$ scores obtained by running Demixer and QuantTB on ART-TBmix1 and ART-TBmix2 datasets. These scores along with the related precision and recall measures are in Supplementary Tables S4 and S5. (C) Performance of the two modes of Demixer on ARTmix data. The *y*-axis of both the top and bottom plots represents the mean RE (see Supplementary Section S1.5), with error bar overall length being twice the standard deviation across the 10 runs of Demixer (each involving a different random seed). The *x*-axis of the top plot corresponds to the number of mixed strains present in ARTmix samples and the *x*-axis of bottom plot correspond to the different combinations of reference and *de novo* strains in the ARTmix samples.

To enable further comparison of a multi-sample analysis method like Demixer with a per-sample-analysis tool like QuantTB, we evaluated both methods on QuantTB benchmark datasets (specifically ART-TBmix{1,2}, which were generated as per the description in the QuantTB publication). The $F1_{\mathrm{avg}}$ score of Demixer is comparable to that of QuantTB for the ART-TBmix1 dataset, whereas better than QuantTB for the ART-TBmix2 dataset (Fig. 4B). Note that the $F1_{\mathrm{avg}}$ scores of Demixer are greater than 0.90 at all coverage levels of ART-TBmix{1,2}. Demixer has relatively lower performance at 10× coverage level in ART-TBmix1, which may be due to the low coverage data not being sufficient to learn the parameters of the probabilistic model underlying Demixer.

We also compared Demixer and QuantTB using the ARTmix dataset consisting of 18 pure and 32 mixed samples in ‘all references known’ mode. In this mode, the SNP-alleles of the strains used to generate the ARTmix samples were revealed in the form of a reference database to both Demixer and QuantTB. As ARTmix includes mixed samples with both two and three strains, we have computed the mean RE for each case by including only the corresponding samples. Though Demixer’s mean RE is slightly higher than that of QuantTB in samples mixed with two strains, they are still comparable as the relative errors are very low (top plot of Fig. 4C).

While QuantTB is a state-of-the-art method for estimating TB strain proportions, it is fully reference-based and cannot identify novel strains. Our Demixer however can identify new strains due to its hybrid (reference-plus-*de novo*) design. To evaluate how well Demixer can identify new strains, we ran it in ‘few references known’ (*de novo*) mode on the ARTmix dataset. In this mode, the SNP-alleles of only five strains are revealed to Demixer. Demixer’s RE in *de novo* mode (bottom plot of Fig. 4C) is, as expected, higher than Demixer in ‘all references known’ mode and QuantTB. However, the RE is at most 0.15, and so Demixer in *de novo* mode can identify strains whose SNP-alleles are not in the reference database with reasonable accuracy.

### 3.4 Demixer detects new mixed samples in Malawi dataset

**Figure 5.**
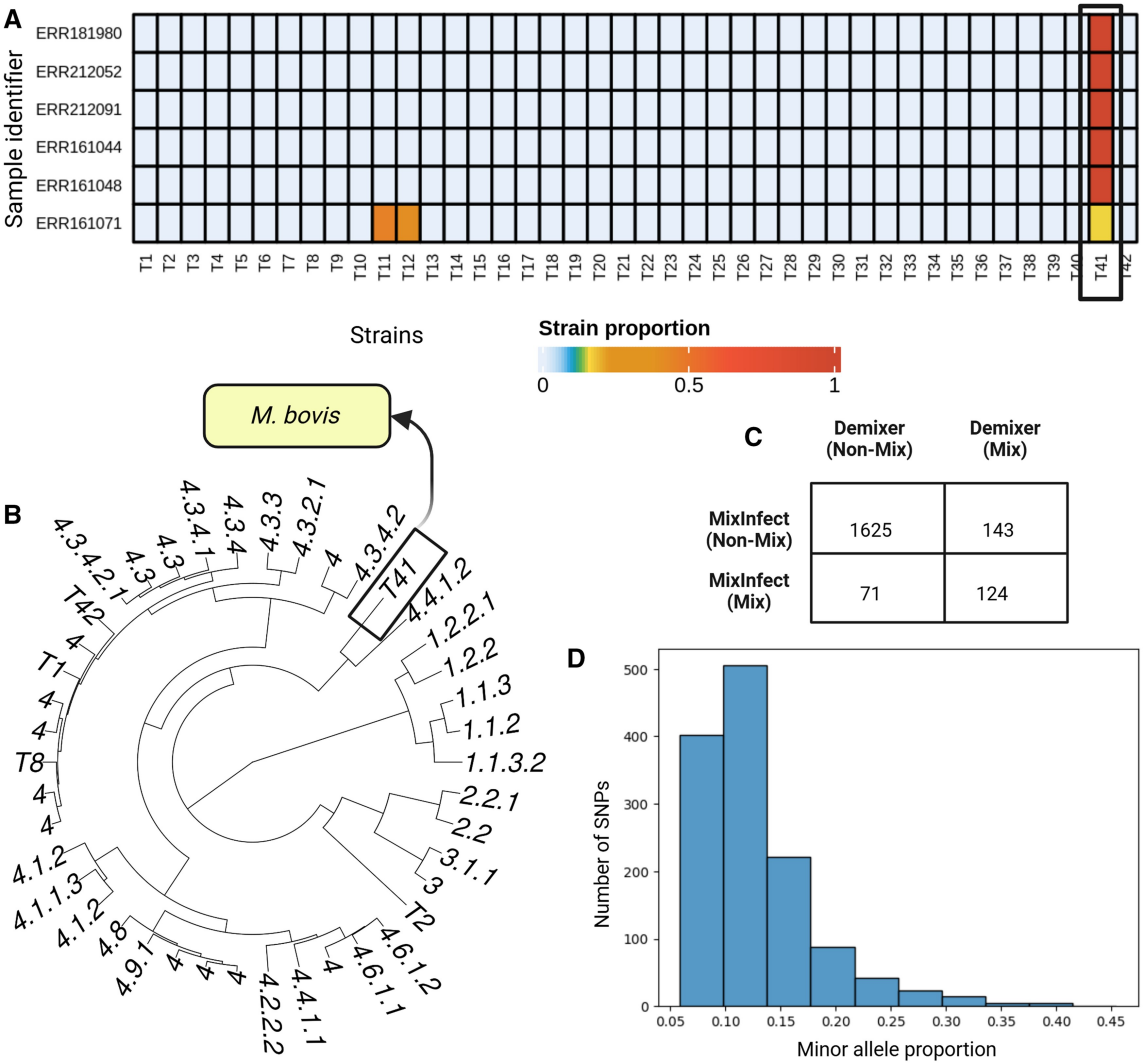
Demixer results on Malawi dataset. (A) The Demixer-estimated strain proportions of samples containing *M. bovis* strains. Here, Demixer is run by hiding the SNP-alleles relevant to *M. bovis* strain. (B) The hierarchical clustering of the inferred strains. The strain number T41 corresponds to the new strain identified by Demixer, which is distant from the lineages 1, 2, and 3. (C) The number of samples identified as mixed and non-mixed by Demixer and MixInfect. (D) The SNP plot of sample ERR161149 identified as mixed by Demixer and non-mixed by MixInfect. The *x*-axis refers to the proportion of the minor alleles in a heterozygous SNP and *y*-axis refers to the frequency of such SNPs with that minor allele proportion.

In the previous section, we have seen how Demixer can detect *de novo* strains within a synthetic dataset. To assess the same in a real-world setting, we consider the Malawi samples comprising major lineages (1–4) of the species *M. tb* and another TB-related species *M. bovis*. We specifically applied Demixer on these samples after hiding the unique SNP-alleles of *M. bovis* strain in the reference database. The SNP-alleles corresponding to 40 strains are found in the sample set, so the model parameter K is set to 42 (see Section 2). TBProfiler identifies five pure *M. bovis* samples and one sample mixed with *M. bovis* and lineage 3 strain in the Malawi dataset. The proportions determined by Demixer for those six samples containing the unmapped strain T41 (Fig. 5A and B) indicate that Demixer can delineate the proportions even after the exclusion of *M. bovis* SNP-alleles from the reference database. The new strain T41 was detected only in the above six *M. bovis* samples, and the strain’s proportion in the five pure *M. bovis* samples were also correctly identified as 100%. The 62 mutations of the T41 strain, as inferred from the Strain-SNP-allele distribution provided by Demixer is in agreement with the reference mutations of *M. bovis* strain. These results indicate that Demixer can infer the mutational profile as well as the proportion of the *de novo* strain (*M. bovis* in this case) accurately.

Next, we applied Demixer to the 1963 Malawi samples after including all the strains in the reference database. Demixer was able to identify known (124) as well as novel (143) mixed infection samples, relative to previously reported mixed samples (Sobkowiak *et al.* 2018) (see Fig. 5C and Supplementary File D2). The previously reported calls were made by MixInfect, a tool similar to SplitStrains in terms of estimating the proportion but not the mutational profile of strains. To highlight an example of a novel mixed infection sample, consider ERR161149—Demixer identifies this sample as a mixture of strains 4.3.4.2.1 (95%) and 4.4.1.2 (5%), whereas MixInfect calls this sample as pure. When checking this sample using a SNP plot (Fig. 5D), we found clear evidence for a mixture of strains (due to the presence of 1309 heterozygous SNPs in the sample with mode $o_{m}$ being equal to 11%). When checking all 143 novel mixed calls using a SNP plot related confidence measure $f_{m}$ (see Section 2), we could classify all but five of them as medium/highly confident mixed calls (Supplementary Fig. S7A); further, their $f_{m}$ values taken together was higher than the $f_{m}$ values of the 71 samples called as mixed by MixInfect but not Demixer (Wilcoxon ranksum test one-sided *P*-value $2.21\times 10^{-25}$, with associated statistic value of 9489; see also Supplementary Fig. S7B). Taken together, our multi-sample analysis method Demixer can detect mixed infection with high sensitivity in samples obtained from a clinical setting.

### 3.5 Demixer infers global frequency of mixed infections and its association to drug resistance

**Figure 6.**
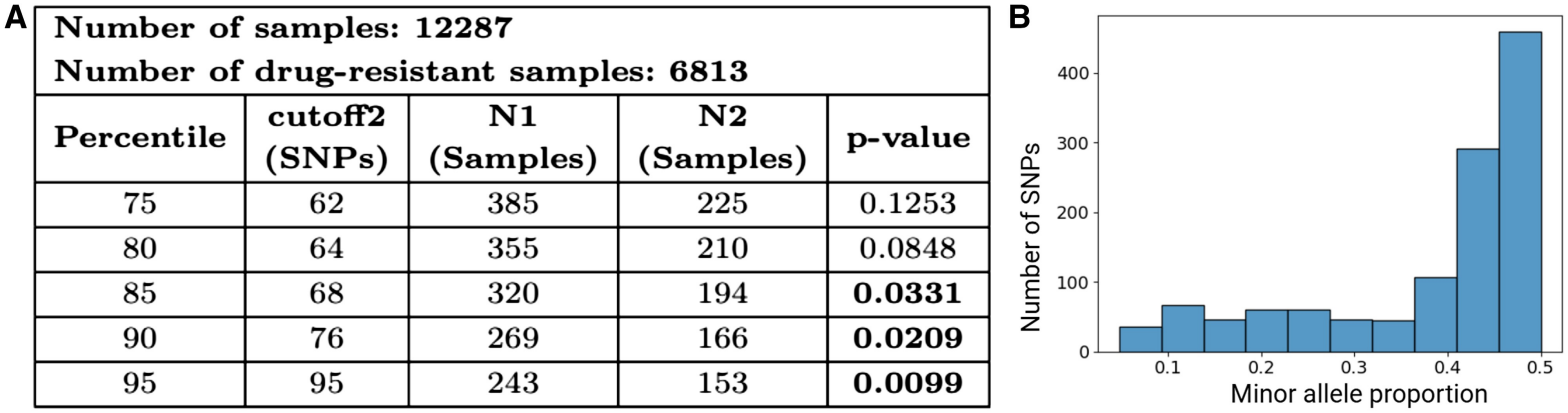
Association between mixed infection and drug resistance in CRyPTIC samples. (A) Hypergeometric test *P*-values to assess the association between drug resistance and mixed infection are shown, using high-confidence mixed infection calls based on different $f_{m}$ and $o_{m}$ thresholds. N1 refers to the number of mixed samples whose $f_{m}>$ cutoff2 and $o_{m}>$ 0.05, and N2 refers to a subset of the samples accounted for in N1 that are also resistant to at least one drug. (B) SNP plot of the mixed infection sample resistant to all the 13 tuberculosis drugs. The interpretation of SNP plot is same as in Fig. 5D. See Supplementary File D3 for the estimated lineages and proportions of all CRyPTIC samples.

To detect mixed infection confidently in clinical settings across the globe and to assess its relation to drug resistance, we applied Demixer to the CRyPTIC dataset and focused only on Demixer’s high-confidence calls (as determined using the $f_{m}$ and $o_{m}$ measures; see Section 2 and Supplementary Fig. S8A). Demixer identified 385 (3.3% of the total 12287) isolates to be mixed infection with high confidence (see Fig. 6A). When testing overlap against the 6813 CRyPTIC isolates resistant to at least one TB drug, we found that 225 of these 385 mixed infection isolates are drug-resistant. This overlap is marginally significant (hypergeometric *P*-value 0.1253). However as shown in Fig. 6A, the statistical association between mixed infection and drug resistance becomes stronger as the stringency for high-confidence mixed infection calls by Demixer are increased progressively (from 75th to 95th percentiles of ${\left\{f_{m}\right\}}_{m=1}^{M}$, with hypergeometric *P*-value .0099 for the most stringent threshold).

In addition to revealing global patterns of mixed infection and its link to drug resistance in the CRyPTIC dataset, Demixer could also reveal useful information about each individual sample. For instance, of the two CRyPTIC samples that are resistant to all 13 drugs, Demixer identified one of them as a pure sample of lineage 2.2.1 and the other as a mixture of strains belonging to lineages 2.2.1 (44%) and 4.2.1 (56%). The SNP plot of the latter mixed sample is shown in Fig. 6B. As another application of Demixer, we also collated its predictions to calculate the percentage of mixed infection samples that are resistant separately to each antituberculous drug (see Supplementary Fig. S8B). Finally, we analysed a small subset of samples from the Malawi dataset using the ϕ obtained by running Demixer on the CRyPTIC dataset, emphasizing the efficacy of the method for real-time analysis (see Supplementary File D4 for the inferred strains and proportions on the Malawi subset). These applications together show that Demixer is a useful tool for predicting mixed infection and assessing its link to drug resistance.

## 4 Discussion

This work presents Demixer, a new probabilistic approach based on topic modeling to aid in the detection of mixed strains present in microbial samples. Such modeling has enabled a multi-sample analysis, which is first of its kind to estimate identity and proportion of mixed strains from WGS data, with comparable or better performance over other approaches. The utilization of information about known strains gives Demixer the advantage of reference-based approaches, whereas the probabilistic modeling along with multi-sample analysis facilitate the identification of *de novo* strains. The parallelization heuristic allows Demixer to scale to large datasets containing 1500 or more samples (see Supplementary Table S7). Demixer has been extensively evaluated on different synthetic benchmarks and real-world datasets. For instance, Demixer employing simple hyperparameters (chosen from a systematic tuning approach using synthetic LDAmix* datasets) is effective in estimating strain proportions on the *in vitro* benchmark and other synthetic datasets. Demixer’s results on the Malawi and CRyPTIC dataset demonstrate the benefits of using prior information in determining the identity of strains in samples obtained from a clinical setup. They also point to a scenario where Demixer parameters learnt from a large dataset like CRyPTIC can be used to infer strains in new samples in a smaller dataset refer Supplementary Section S1.6.5. Demixer is generalizable and can be applied to other bacterial or microbial species as well.

The strain estimations by our Demixer and the previously published MixInfect methods are agreeable in 89% of the samples, and the disagreements could be attributed to differences in the variant calling or preprocessing workflows, and the mixed infection detection methodology (e.g. single- versus multi-sample analysis). The detection of 3.1% mixed infections (after postprocessing using default cutoffs) in the CRyPTIC dataset is not too far from the 1.5% mixed infections reported in (CRyPTIC Consortium, 2022b). Additionally, Demixer identified four *Mycobacterium orygis* samples, which were not reported in (CRyPTIC Consortium, 2022b). When comparing Demixer with other methods on real-world datasets, we resorted to SNP plots and associated measures ($f_{m}$ values) to validate the strains predicted by a method. We used these proxy validation measures to compare methods, since ground-truth experimentally validated mixed infection datasets (like the *in vitro* dataset) are scarce. Once more such datasets become available, we can better address the uncertainty around whether the presence of SNPs from two different strains in a sample truly indicates mixed infection, and if so, what is the minimum number of such SNPs required to be present.

We wanted to compare Demixer with popular word-embedding-based models of microbial DNA sequences. But extensive literature survey showed that existing embedding-based models do not directly address the problem of detecting mixed infection. Instead, embedding-based deep learning methods, such as DeepMicrobes (Liang *et al.* 2020) and DNABERT-2 (Zhou *et al.* 2023), perform taxonomic (genus/species-level) classification of short meta-genomic reads. Hence, we adapted these methods to perform strain-level classification, which is a key step in detecting mixed infection (see Supplementary Section S1.6.3 and Fig. S9) and compared the adapted methods to Demixer. As such, this is not a direct one-to-one comparison due to the adaptation step, however this is the best we could do to assess Demixer alongside certain popular deep learning models. Results from this comparative analyses is summarized below.

- DeepMicrobes and DNABERT-2 demonstrated comparable performance to Demixer in identifying the majority strains present in *in vitro* samples (see Supplementary Table S8). However, Demixer also accurately identifies their respective proportions with a relative error of 0.03, whereas embedding-based models have a relative error greater than 0.2 (shown in Supplementary Fig. S10).
- Demixer can also learn the mutational profiles of both seen and unseen strains as part of its multi-sample inference procedure, unlike embedding-based models which cannot yield such profiles. These profiles make Demixer’s output more interpretable.

These results imply how Demixer is well-suited to delineate the strains in a mixed infection sample, and highlight the challenges involved in adapting a deep learning model to do the same.

Huang *et al.* (2022) emphasize the importance of identifying the lineages of strains in an individual before initiating the treatment, as mixed infections due to nontuberculous mycobacteria (NTM) and *M. tb* can impact treatment outcomes. Therefore, a reference database consisting only of *M. tb* lineages may not be sufficient to identify mixed infections due to *M. tb* and NTM. The increasing trend of TB caused by *M. bovis*, with its natural resistance to pyrazinamide, further complicates matters (Bobadilla-del Valle *et al.* 2015). Demixer has the potential to identify such NTM strains even if they are not present in the reference database, as they are dissimilar from *M. tb* sequences. Our results on synthetic and real-world datasets demonstrated the effectiveness of Demixer in disentangling dissimilar *de novo* strains. The importance of developing new techniques to identify novel strains has also been highlighted by the authors of the recently published Fastlin tool (Derelle *et al.* 2023).

Regarding limitations of Demixer, one potential concern with our approach is that a model that is tuned on one dataset (LDAmix* in our case) may not work well on other datasets; however this was not the case in the applications of Demixer we have tried till now. Another concern could be about the completeness of the reference database that we use with Demixer, even though we have taken care to choose a widely-used reference database. For instance, the latest subtypes of lineage 2 are still missing in the reference database, and this could mislead inferences of Demixer on samples containing these subtypes. Demixer could also be misled about closely related strains that have insufficient number of SNP-alleles separating them in the reference database. This underscore the need for efforts to continuously update the reference database used with Demixer. A final concern is that, like other LDA models, Demixer requires careful selection of the number of strains K to achieve meaningful and interpretable results. A high K value may mislead the model to declare a sub-pattern present in a lineage as a new strain, thereby leading to unnecessary fragmentation of strains. We carefully choose K using the $K=K^{\prime }+2$ heuristic, where $K^{\prime }$ captures only the reference strains whose SNP-alleles are in the input samples and the extra 2 enables detection of *de novo* strains. Through this heuristic and via postprocessing (hierarchical clustering) of sub-strains into fewer strains, we mitigated the fragmentation issue in complex real-world TB datasets. Future work could focus on improving Demixer by developing better heuristics for selecting K and assigning weights to reference SNP-alleles (since Demixer performance can be sensitive in certain datasets to the current weighting heuristics; see Supplementary Fig. S11).

Demixer represents a novel systematic approach for delineating the mixed strains in microbial samples, as demonstrated here in its applications to diverse datasets. These results encourage different future extensions of Demixer. One interesting future extension could be to model the hierarchical relationship between the inferred strains directly into the underlying SNP-LDA model of Demixer and see if the performance is further improved. In the current implementation, Demixer incorporates an input lineage tree (and the assumption that SNP-alleles present in a parent strain are also present in descendant strains) to infer hierarchically related strains present in a sample. Another fruitful extension of Demixer could be to analyse meta-genomic reads for strain-level identification (Anyansi *et al.* 2020b). Demixer in its current and extended forms holds promise to reveal new insights into mixed infection diagnosis and treatment.

## Supplementary Material

btaf139_Supplementary_Data

## Data Availability

As mentioned in Supplementary Information, Suppl Data Files are available at this link: https://drive.google.com/drive/folders/1P_OX_MbZ6QFN9Amyl2eGMBr1ySY6yNWu. The Suppl data, code and vcf files (of in vitro, synthetic and real-world datasets) have also been archived at Zenodo (doi:10.5281/zenodo.15074330). As mentioned before, all code and associated data relevant to Demixer are available at https://github.com/BIRDSgroup/Demixer.
