# Supplementary material for: Demixer: a probabilistic generative model to delineate different strains of a microbial species in a mixed infection sample
Source: Bioinformatics. 2025 Apr 3;41(4):btaf139. doi: 10.1093/bioinformatics/btaf139 (PMC12011361; doi:10.1093/bioinformatics/btaf139)
Supplement: btaf139_Supplementary_Data [file btaf139_supplementary_data.zip › Supplementary_Information_Demixer.pdf]

Supplementary Information

for the manuscript Demixer: A probabilistic generative model to delineate different strains of a microbial species in a mixed infection sample

1 Supplementary Methods **1**
- 1.1 Background on LDA model 1
- 1.2 From a reference database to global/sample-specific dictionaries 1
- 1.3 Demixer description - Additional information 2
  - 1.3.1 Preprocessing 2
  - 1.3.2 Estimation of strain proportion 2
  - 1.3.3 Postprocessing/interpretation steps 5
- 1.4 Datasets used in the analysis 6
- 1.5 Evaluation metrics 7
- 1.6 Other aspects of Demixer 8
  - 1.6.1 Comparison of Demixer and traditional LDA 8
  - 1.6.2 Model selection and hyperparameter tuning 8
  - 1.6.3 Comparison of Demixer with embedding-based methods 8
  - 1.6.4 Implementation details 9
  - 1.6.5 Using Demixer for analysing new sample(s) 10

2 Supplementary Algorithms **12**

3 Supplementary Tables **14**

4 Supplementary Figures **16**

5 Supplementary Files **20**

1. Supplementary Methods

1.1. Background on LDA model

LDA, proposed by Blei et al. (Blei et al. 2003), is an unsupervised approach that is widely used in text mining to infer underlying topics in a corpus of documents. It belongs to the family of latent variable models, whose basic principle to model a given system is to employ a complex hidden mechanism (latent generative process) to link the unobserved (latent) variables to the observed variables in the system. Specifically, as shown next, LDA assumes that each document is generated using a mixture of K topics, and knowing the topic (latent variable z) assigned to each word w in the document could facilitate the estimation of document-topic distribution $\boldsymbol{\theta}$ and word-topic distribution $\boldsymbol{\phi}$.

- For each document d, generate $\boldsymbol{\theta}_d \sim \text{Dirichlet}(\alpha)$.
- For each topic k, generate $\boldsymbol{\phi}_k \sim \text{Dirichlet}(\beta)$.
- To generate n-th word $w_{n,d}$ in each document d:
  - Generate $z_{n,d} \sim \text{Multinomial}(\boldsymbol{\theta}_d)$.
  - Generate $w_{n,d} \sim \text{Multinomial}(\boldsymbol{\phi}_{z_{n,d}})$.

Here, α and β are the hyperparameters of the LDA model; $z_{n,d}$ is the latent variable capturing the topic assigned to word $w_{n,d}$ at n^{th} position in document d. The generate term above refers to drawing/choosing/sampling a value for a random variable from the specified distribution. The model parameters are learnt from data w using variational or Gibb's sampling based inference algorithms.

Recently, LDA has been used in bioinformatics applications involving large biological datasets analogous to LDA documents and words. Liu et al. (Liu et al. 2016) have categorized such applications into three types: clustering, classification, and feature extraction of biological data using the generative principle of LDA. Classic examples include gene sequence classification, metagenomic binning, and learning protein functions. As mentioned in main text, most applications use published LDA models directly to address specific challenges (Liu et al. 2016). We had to modify and extend the LDA model to capture the reads assigned to the four alleles of a SNP (which has different semantics than words in a text); and we also propose a parallelization heuristic to speedup the CGS inference algorithm on our Demixer SNP-LDA model.

1.2. From a reference database to global/sample-specific dictionaries

The reference database of Demixer is constructed using the 7684 barcoding SNP-alleles identified by Napier et al. (Napier et al. 2020) to distinguish 91 TB lineages and sublineages (all of which are referred to simply as strains for convenience; the SNP-alleles in the reference database are also called as reference SNP-alleles). As mentioned in the main text (and explained later in Suppl Section 1.3.3), we have access to a lineage tree that represents the hierarchical (ancestor-descendant) relationship between all the strains (lineages/sublineages) in the reference database. We now derive two types of databases, RDB1 and RDB2.

RDB1 includes only the unique SNP-alleles of each strain - a SNP-allele is called unique for a strain j if it is present in that strain and additionally absent in all other non-descendant strains of strain j in the reference database. Note that these unique SNP-alleles exclusively identify a strain (excepting all its descendants). RDB1 can be viewed as a binary matrix of dimension $7684 \times L$, where L is the number of

strains. Note that, more the number of unique SNP-alleles for a specific strain in RDB1, the more information we've on this strain in the reference database, making it less likely for Demixer to miss the identification of that strain.

RDB2 consists of all SNP-alleles recorded in the reference database (including both unique and non-unique SNP-alleles of strains). It is also represented as a matrix and its entry RDB2$[(n, v), j] = 1$ if SNP-allele (n, v) is present in strain j or any ancestor of strain j. In other words, the SNP-alleles of each strain is propagated down to all its descendant strains; with the assumption being that a SNP-allele in a strain is also present in all its children and descendant strains (for example, if A100T belongs to strain 1.2.1, then A100T will also belong to all its descendants 1.2.1.x). Using these RDB* databases, we derive the dictionaries GD and SD$_m$; please refer Algorithm 1 for the usage of these dictionaries in the parallelized CGS algorithm.

To construct the global dictionary (GD), we start with the pruning of the lineage tree. The rows corresponding to SNP-alleles that are absent in all input samples are removed from RDB1, followed by the removal of columns/strains with less than 5 non-zero entries - the SNP-alleles and strains removed during this process are also removed from RDB2, and the resulting RDB2' is used to create the global dictionary (GD). In this dictionary, each key corresponds to the SNP-allele (n, v), and the related value is the list of all strains j such that RDB2'$[(n, v), j]$=1.

To construct the sample-specific dictionary (SD$_m$), we follow the same procedure as in the above paragraph, but we start with the removal of SNP-alleles that are absent in sample m alone (instead of those absent in all input samples); and also remove only the columns/strains with 0 non-zero entries (instead of less than 5 non-zero entries as before).

1.3. Demixer description - Additional information

This section provides additional detailed information on the different steps of our Demixer method, by expanding on the description of Demixer given in the main text.

1.3.1. Preprocessing

Preprocessing involves different steps to process the FASTQ files containing raw reads of WGS samples to generate the Sample-SNP-allele matrix $\boldsymbol{S}$.

From WGS reads to genetic variants: A preprocessing pipeline for extracting the variants from WGS reads of samples has been developed. The quality of the raw reads is analyzed using the FastQC (version 0.11.9) (Andrews et al. 2017) tool. Burrows-Wheeler Alignment (BWA) tool (version 0.7.17) (Li and Durbin 2009) is used to map the raw reads in the sample against the H37Rv reference genome (NCBI Reference Sequence NC_000962.3). The output of BWA is subject to de-duplication and BAM file generation using the GATK tool (Genome Analysis Toolkit, version 4.1.8.0). From the resulting .bam file, FreeBayes (version 1.3.6) is used to call variants and generate the .vcf file (with –gvcf and –monomorphic options enabled, if the number of samples ≤ 100). The .vcf files of individual samples are then merged using bcftools (version 1.14) (Danecek et al. 2021). The above pipeline is used to preprocess all the synthetic and benchmark datasets. As per standard practice, variants from PE/PPE gene regions are removed, and only SNPs from the coding regions are extracted for further analysis of the TB isolates (Sobkowiak et al. 2018). In addition, the following filters are applied to individual datasets:

- ART-TBmix1, ART-TBmix2: Exclusion of variants with Allele Count (AC) == 0
- *in vitro*: Exclusion of variants with Depth of coverage (DP) < 5, Genotype Quality (GQ) < 20, and Minor Allele Frequency (MAF) ≤ 0

For the Malawi dataset (as number of samples > 100), a second round of variant calling is executed to call variants specifically at the positions in the merged .vcf file for each sample, which are then finally merged across all samples to get a single .vcf file. We use dataset-specific MAF cutoff values to adapt to the different sizes of real-world datasets (Malawi - 0.002 and CRyPTIC - 0.0002; see Section 2.3 for details of these datasets). For the Malawi dataset, we applied the MAF filter both before (to speedup the process) and after the second variant calling on the merged vcf files. In the case of CRyPTIC, we used the processed .vcf files (with two rounds of variant calling) of individual samples obtained from their repository to get the final merged .vcf file and then applied the MAF cut-off.

Construction of Sample-SNP-allele matrix: The distinctive characteristics of different strains of a cellular organism are often related to specific genotypic features that serve as unique identifiers. One such genetic marker, SNPs within the DNA sequence, is widely used to distinguish the different strains reliably. A detailed analysis of the distribution of different alleles (A, C, G, T) of such SNPs present in a sample is required to detect mixed infection and delineate the proportion of the microbial strains in the sample (Sobkowiak et al. 2018). This SNP-allele distribution is provided by the WGS reads obtained from a sample, with each read supporting a SNP-allele combination similar to a word in text LDA analysis (Zhao et al. 2016). The number of reads supporting each SNP-allele combination for all samples captures sufficient information for the model to learn the underlying strains, and we refer to this read counts matrix as the Sample-SNP-allele matrix $\boldsymbol{S}$. The $\boldsymbol{S}$ matrix is obtained from the single .vcf file mentioned above by including only the SNPs (i.e., by ignoring other variants like insertions and deletions). Very small entries in the $\boldsymbol{S}$ matrix (specifically any entry with less than 6 supporting reads) are made zero to mitigate noise from sequencing errors.

1.3.2. Estimation of strain proportion

i) **CGS inference and parameter estimation:** The probability of assigning a strain to a read (i.e., sampling $z_{m,n,r}$) given the strain assignment of all other reads (denoted by $z_{-(m,n,r)}$) is derived (Carpenter 2010) as below:

$$\begin{aligned}\mathbb{P}(z_{m,n,r} = k \mid z_{-(m,n,r)}, w, \alpha, \beta) &\propto \mathbb{P}(z_{m,n,r} = k, z_{-(m,n,r)}, w \mid \alpha, \beta)\\ &= \mathbb{P}(z, w \mid \alpha, \beta) \qquad (1)\end{aligned}$$

Now, we derive $\mathbb{P}(z, w \mid \alpha, \beta)$ using our model structure (i.e., conditional independence assumptions implied by the model) as follows:

$$
\begin{aligned}
\mathbb{P}(z, w \mid \alpha, \beta) &= \mathbb{P}(z \mid \alpha) \times \mathbb{P}(w \mid z, \beta) \\
&= \int \mathbb{P}(\boldsymbol{\theta} \mid \alpha)\ \mathbb{P}(z \mid \boldsymbol{\theta})\ \mathrm{d}\boldsymbol{\theta} \times \int\ \mathbb{P}(\boldsymbol{\phi} \mid \beta)\ \mathbb{P}(w \mid \boldsymbol{\phi}, z)\ \mathrm{d}\boldsymbol{\phi} \\
&= \int \prod_{m=1}^{M} \mathbb{P}(\boldsymbol{\theta}_m \mid \alpha) \prod_{n=1}^{N} \prod_{r=1}^{R} \mathbb{P}(z_{m,n,r} \mid \boldsymbol{\theta}_m)\ \mathrm{d}\boldsymbol{\theta} \times \int \prod_{n=1}^{N} \left(\prod_{k=1}^{K} \mathbb{P}(\boldsymbol{\phi}_{k,n} \mid \beta_n) \right) \prod_{m=1}^{M} \prod_{r=1}^{R} \mathbb{P}(w_{m,n,r} \mid \boldsymbol{\phi}_{z_{m,n,r},n})\ \mathrm{d}\boldsymbol{\phi} \\
&= \left(\prod_{m=1}^{M} \int \mathbb{P}(\boldsymbol{\theta}_m \mid \alpha) \prod_{n=1}^{N} \prod_{r=1}^{R} \mathbb{P}(z_{m,n,r} \mid \boldsymbol{\theta}_m)\ \mathrm{d}\boldsymbol{\theta}_m \right) \times \left(\prod_{n=1}^{N} \int \left(\prod_{k=1}^{K} \mathbb{P}(\boldsymbol{\phi}_{k,n} \mid \beta_n) \right) \prod_{m=1}^{M} \prod_{r=1}^{R} \mathbb{P}(w_{m,n,r} \mid \boldsymbol{\phi}_{z_{m,n,r},n})\ \mathrm{d}\boldsymbol{\phi}_n \right)
\end{aligned} \tag{2}
$$

Letting $V = \{A, C, G, T\}$, expanding the discrete distribution $\mathbb{P}(w \mid \boldsymbol{\phi}, z)$ using the indicator function $\mathbb{1}$ (defined in the main text), and grouping the resulting terms by $q \in V$ and by the strains k as in (Carpenter 2010), we can simplify some of the terms in the above Equation as follows:

$$
\begin{aligned}
\prod_{m=1}^{M} \prod_{r=1}^{R} \mathbb{P}(w_{m,n,r} \mid \boldsymbol{\phi}_{z_{m,n,r},n}) &= \prod_{m=1}^{M} \prod_{r=1}^{R} \prod_{q \in V} \boldsymbol{\phi}_{z_{m,n,r},n,q}^{\mathbb{1}(w_{m,n,r}==q)} \\
&= \prod_{m=1}^{M} \prod_{r=1}^{R} \prod_{q \in V} \prod_{k=1}^{K} \boldsymbol{\phi}_{k,n,q}^{\mathbb{1}(w_{m,n,r}==q \text{ AND } z_{m,n,r}==k)} \\
&= \prod_{q \in V} \prod_{k=1}^{K} \boldsymbol{\phi}_{k,n,q}^{\sum_{m=1}^{M} \sum_{r=1}^{R} \mathbb{1}(w_{m,n,r}==q \text{ AND } z_{m,n,r}==k)} \\
&= \prod_{k=1}^{K} \prod_{q \in V} \boldsymbol{\phi}_{k,n,q}^{\boldsymbol{C}_{k,*,n,q}}
\end{aligned} \tag{3}
$$

Similarly,

$$
\prod_{n=1}^{n} \prod_{r=1}^{R} \mathbb{P}(z_{m,n,r} \mid \boldsymbol{\theta}_m) = \prod_{k=1}^{K} \boldsymbol{\theta}_{m,k}^{\boldsymbol{C}_{k,m,*,*}} \tag{4}
$$

Substituting Equations 3, 4 into Equation 2, we get:

$$
\begin{aligned}
\mathbb{P}(z, w \mid \alpha, \beta) &= \left(\prod_{m=1}^{M} \int \mathbb{P}(\boldsymbol{\theta}_m \mid \alpha) \prod_{k=1}^{K} \boldsymbol{\theta}_{m,k}^{\boldsymbol{C}_{k,m,*,*}}\ \mathrm{d}\boldsymbol{\theta}_m \right) \times \left(\prod_{n=1}^{N} \int \left(\prod_{k=1}^{K} \mathbb{P}(\boldsymbol{\phi}_{k,n} \mid \beta_n) \right) \prod_{k=1}^{K} \prod_{q \in V} \boldsymbol{\phi}_{k,n,q}^{\boldsymbol{C}_{k,*,n,q}}\ \mathrm{d}\boldsymbol{\phi}_n \right) \\
&= \left(\prod_{m=1}^{M} \int \mathbb{P}(\boldsymbol{\theta}_m \mid \alpha) \prod_{k=1}^{K} \boldsymbol{\theta}_{m,k}^{\boldsymbol{C}_{k,m,*,*}}\ \mathrm{d}\boldsymbol{\theta}_m \right) \times \left(\prod_{n=1}^{N} \prod_{k=1}^{K} \int \mathbb{P}(\boldsymbol{\phi}_{k,n} \mid \beta_n) \prod_{q \in V} \boldsymbol{\phi}_{k,n,q}^{\boldsymbol{C}_{k,*,n,q}}\ \mathrm{d}\boldsymbol{\phi}_{k,n} \right) \\
&= \left(\prod_{m=1}^{M} \int \frac{1}{B(\alpha)} \prod_{k=1}^{K} \boldsymbol{\theta}_{m,k}^{\alpha_k - 1 + \boldsymbol{C}_{k,m,*,*}}\ \mathrm{d}\boldsymbol{\theta}_m \right) \times \left(\prod_{n=1}^{N} \prod_{k=1}^{K} \int \frac{1}{B(\beta_n)} \prod_{q \in V} \boldsymbol{\phi}_{k,n,q}^{\beta_{n,q} - 1 + \boldsymbol{C}_{k,*,n,q}}\ \mathrm{d}\boldsymbol{\phi}_{k,n} \right) \\
&= \left(\prod_{m=1}^{M} \frac{B\left(\{\alpha_k + \boldsymbol{C}_{k,m,*,*}\}_{k=1}^{K} \right)}{B\left(\{\alpha_k\}_{k=1}^{K} \right)} \right) \times \left(\prod_{n=1}^{N} \prod_{k=1}^{K} \frac{B\left(\{\beta_{n,q} + \boldsymbol{C}_{k,*,n,q}\}_{q \in V} \right)}{B\left(\{\beta_{n,q}\}_{q \in V} \right)} \right)
\end{aligned}
$$

In the last two steps above, we had expanded the Dirichlet distributions $\mathbb{P}(\boldsymbol{\theta}_m \mid \alpha)$ and $\mathbb{P}(\boldsymbol{\phi}_{k,n} \mid \beta_n)$ and used the normalizing constants of the Dirichlet distribution (the multivariate beta function $B(.)$). Substituting above expression into Equation 1, and simplifying further by expanding the beta functions and dropping constant terms as in (Carpenter 2010), yields the following CGS update Equation:

$$
\mathbb{P}(z_{m,n,r} = k \mid z_{-(m,n,r)}, w, \alpha, \beta) \propto (\boldsymbol{C}_{k,m,*,*}^{-(m,n,r)} + \alpha_k) \times \frac{(\boldsymbol{C}_{k,*,n,v}^{-(m,n,r)} + \beta_{n,v})}{(\boldsymbol{C}_{k,*,n,*}^{-(m,n,r)} + \beta_{n,*})} \qquad (\text{here, } w_{m,n,r} = v) \tag{5}
$$

Once the strain identifier $z_{m,n,r}$ is sampled using Equation 5 above, its sampled value is used to update the counts $\boldsymbol{C}_{k,m,*,*}$ and $\boldsymbol{C}_{k,*,n,v}$ (recall $w_{m,n,r} = v$). The unnormalized $\boldsymbol{\theta}$ and $\boldsymbol{\phi}$ can then be determined from the above updated counts as:

$$\boldsymbol{\theta}_{m,k} = \boldsymbol{C}_{k,m,*,*}$$
$$\boldsymbol{\phi}_{k,n,q} = \boldsymbol{C}_{k,*,n,q}$$

When calculating KL divergence measures that involve inferred $\boldsymbol{\phi}$ distributions, we add a pseudocount of $\beta_{n,q}$ to the estimate of $\boldsymbol{\phi}_{k,n,q}$ above to avoid infinite KL divergence values.

ii) **Heuristic Strategies:** This section describes the different heuristics employed for optimizing the SNP-LDA model.

Initialization heuristics: The SNP-LDA model may struggle to infer the proportions of minority strains that are mixed in very low frequencies in a sample. To address this issue and thereby enhance SNP-LDA model's ability to precisely detect the Sample-Strain distribution, we propose two alternative methods to initialize the model parameters and latent variables.

- **NMF SNP-LDA:** This approach uses the output of NMF to initialize the strain of each read, instead of a random strain initialization described above. NMF is a widely used algorithm that factorizes a non-negative matrix into a product of two non-negative matrices to reveal certain (K') underlying latent components as follows:

$$\boldsymbol{X}_{M'\times N'} \simeq \boldsymbol{Y}_{M'\times K'}\boldsymbol{Z}_{K'\times N'}$$

In our application, the observed data $\boldsymbol{X}$ is the Sample-SNP-allele matrix $\boldsymbol{S}_{M\times N\times 4}$ (collapsed into a 2D matrix of size $M \times 4N$), the inferred matrix $\boldsymbol{Y}$ represents the Sample-Strain distribution $\boldsymbol{\theta}_{M\times K}$, and finally the other inferred matrix $\boldsymbol{Z}$ refers to the Strain-SNP-allele distribution $\boldsymbol{\phi}_{K\times N\times 4}$ (collapsed into a $K \times 4N$ sized 2D matrix). We used the NMF implementation from Python's scikit-learn library (Pedregosa et al. 2011), and chose Non-negative Double Singular Value Decomposition to initialize the NMF procedure.

As NMF is a simpler and efficient non-probabilistic approach, we apply it to factorize $\boldsymbol{S}$ into $\boldsymbol{\theta}$ and $\boldsymbol{\phi}$ matrices, which we then use to initialize/sample the strains of reads. In detail, once we condition upon $\boldsymbol{\theta}$ and $\boldsymbol{\phi}$, the random variables pertaining to different reads in our model become independent; hence we can sample each $z_{m,n,r}$ independently using the observed $w_{m,n,r}$ like so:

$$\begin{aligned}&\mathbb{P}(z_{m,n,r} = k \mid z_{-(m,n,r)}, w, \boldsymbol{\theta}, \boldsymbol{\phi}, \alpha, \beta)\\ &\quad = \mathbb{P}(z_{m,n,r} = k \mid w_{m,n,r}, \boldsymbol{\theta}, \boldsymbol{\phi}) \propto \mathbb{P}(z_{m,n,r} = k, w_{m,n,r} \mid \boldsymbol{\theta}, \boldsymbol{\phi})\\ &\quad = \mathbb{P}(z_{m,n,r} = k \mid \boldsymbol{\theta_m})\,\mathbb{P}(w_{m,n,r} \mid \boldsymbol{\phi}_{k,n})\end{aligned}$$

- **Hybrid SNP-LDA:** In this approach, the strain of a read with a mutation that uniquely identifies a known/reference strain is initialized to that strain, and all other reads are initialized to a random strain. This reference-based approach (see Figure 1A.2 in main text) utilizes information about known strains during the initialization and iterative CGS phases of the algorithm to detect other minority strains more effectively; and is inspired by earlier topic-modeling works that used seed words capturing the underlying corpus to better detect rare topics (Jagarlamudi et al. 2012; Wood et al. 2017). The premise here is that if information about most of the strains is known, then the proportion of the unknown minority strains can also be detected accurately.

  We consider the barcoding SNP-alleles identified in (Napier et al. 2020) that discriminates nearly 91 *M. tb* strains as reference SNP-alleles. The reference SNP-alleles identified in this work are also incorporated into TBProfiler. Each of these reference SNP-alleles is a mutation that uniquely identifies the corresponding reference strain. The reference SNP-alleles present in each sample, the corresponding reference strains, and the hierarchical relationship between these strains are all utilized to assign strain identifiers to reads, both during initialization phase and during the iterative CGS inference phase (see details in Suppl Section 1.2 and Algorithm 1 in Supplementary Information). Please note that hybrid SNP-LDA can also be run with other reference databases, which may include both unique and non-unique reference SNP-alleles (see Suppl Section 1.2), but we assume a sufficient number of unique reference SNP alleles are present to reliably identify strains.
- **Summary of SNP-LDA variants, and hyperparameter choices:** We've seen three variants of SNP-LDA so far, and one more can arise from a combination of the two initialization heuristics. These four variants can be categorized into two reference-free and two reference-based (also known as hybrid) variants as follows: (i) SNP-LDA – a vanilla variant that initializes the strain of each read randomly; (ii) NMF SNP-LDA (also denoted (NMF, SNP-LDA)) – variant that initializes the strains of reads in the SNP-LDA model using the NMF outputs; (iii) (non-NMF) Hybrid SNP-LDA - a reference-based variant that initializes the strain of reads containing reference mutations/SNP-alleles to the corresponding reference strain and other reads randomly; and (iv) (NMF, hybrid SNP-LDA) – another reference-based variant that initializes the strain of reads containing mutations of a user-revealed reference strain p to p itself and other reads using the NMF outputs (this variant has only limited application in synthetic benchmarks where a reference strain in a sample is revealed, and the benchmark task is to find the remaining *de novo* strains in the sample and all strain proportions).

  Across the SNP-LDA model variants above, we employed 5 different hyperparameter combinations $(1, 0.01)$, $(0.01, A)$, $(0.01, B)$, $(A, 0.01)$ and $(B, 0.01)$. The first element in each set corresponds to the α hyperparameter and the second element to the β hyperparameter (see Suppl Section 1.6.2 for details of A and B). We used synthetic datasets LDAmix1 and LDAmix2 (see Section 2.3 and Suppl Section 1.4 for the description of datasets) to select the best model variant and hyperparameter configuration. We also considered a baseline approach, NMF-only, where NMF outputs are the final estimates of $\boldsymbol{\phi}$ and $\boldsymbol{\theta}$.

Heuristic for choosing $\boldsymbol{K}$: For a given input dataset of samples, the default Demixer (hybrid SNP-LDA) uses reference mutations in these samples to determine the total number of strains K. Specifically, K is set to the number of reference strains whose unique mutations (SNP-alleles) are present in the input samples (denoted K'), plus 2 additional strains to facilitate the detection of new strains. We use 2 as the default additional value under the assumption that the reference database is up-to-date and not too many new strains would have evolved considering the slower growth rate of TB bacteria (Gordon and Parish 2018). The number of additional strains can be adjusted

accordingly when applying Demixer to other microbial species. We use this $K = K' + 2$ heuristic for all our analyses, with the exception of model hyperparameter tuning where we have set $K = 3$ or $K = 4$ depending on the dataset characteristics. Note that hyperparameter tuning experiments involves some reference-free SNP-LDA variants, which do not have access to the reference database to calculate K'; hence we hard-coded the values of K for these experiments. In general, K for reference-free (non-hybrid) variants of SNP-LDA or NMF-only approach can be set by trying different values of K and choosing the one that performs best on an evaluation metric of interest.

Weight heuristic: In a dataset with large number of samples, the number of reference SNP-alleles detected in a sample m (denoted A_m) is likely to be a small fraction of the total number of SNP-alleles (called in all the samples, and estimated as N assuming that majority of the N SNPs are non-reference with single allele). To handle this imbalance, we give reference SNP-alleles a higher weight (Wilson and Chew 2010) based on the intuition that the aggregate sum of the read count of reference SNP-alleles should be similar to that of non-reference SNP-alleles. This weight (of at least 2 for reference SNP-alleles) is formulated as:

$$\boldsymbol{wt}_{n,v} = \begin{cases} \max\{2, \left\lfloor \frac{(N'-A)/K}{A/D} \right\rfloor + 1\} & \text{if } (n,v) \text{ is a reference SNP-allele} \\ 1, & \text{otherwise} \end{cases}$$

where $A = \frac{1}{M}\sum_{m=1}^{M} A_m$ is the average number of reference SNP-alleles per sample, and $N' = (\text{Number of SNP-alleles})/2 \approx N$ (as most SNPs are biallelic). The constant D is set by default to 3, which is derived empirically through the execution of our method on *in vitro* dataset across a range of constant values from 0 to 10, as it yielded a relative error that is comparable with the errors estimated using other values of D (see Suppl Table S2). The weights determined using the above formula for the different datasets used in our study are shown in Suppl Table S3. The CGS update formula, in Equation 2 in main text, upon incorporating the above weights becomes:

$$\begin{aligned} &\mathbb{P}(z_{m,n,r} = k \mid z_{-(m,n,r)}, w, \alpha, \beta) \\ &\propto (\alpha_k + \sum_{n',v'} \boldsymbol{wt}_{n',v'} \boldsymbol{C}^{-(m,n,r)}_{k,m,n',v'}) \cdot \frac{(\boldsymbol{wt}_{n,v} \boldsymbol{C}^{-(m,n,r)}_{k,*,n,v} + \beta_{n,v})}{(\sum_{v'} \boldsymbol{wt}_{n,v'} \boldsymbol{C}^{-(m,n,r)}_{k,*,n,v'} + \beta_{n,v'})} \end{aligned} \tag{6}$$

where $v = w_{m,n,r}$.

Parallelization heuristic: Since the running time of a CGS iteration can be quite high when the number of samples and reads are large, we tried parallelizing a CGS iteration. The "delayed count update" strategy used in an earlier WarpLDA model (Chen et al. 2016) can be used to parallelize CGS at the sample level. This delayed update CGS also enables assignment of strains to all reads of a SNP in a single step (using multinomial sampling based on the above Equation 6, but using C instead of $C^{-(m,n,r)}$), which is more efficient than sampling the strains of each read sequentially over multiple steps (using Equation 6 as is). Our implementation of this parallel (delayed update) CGS and multinomial sampling heuristics is illustrated in Suppl Figure S2, and described in Algorithm 1 in Supplementary Information. These adaptations significantly improved the running time of one iteration of the CGS algorithm for processing all samples from $O(MNRK)$ to $O(\frac{MNRK}{P})$, where P is the available number of processing cores (note $P \leq M$ as our parallelization is at the sample level). The space utilization of Demixer is $O(MNK)$. The empirical memory usage and running time of Demixer for *in vitro* and real-world datasets are shown in Suppl Table S7.

1.3.3. Postprocessing/interpretation steps

The different steps involved in postprocessing the strains inferred by Demixer are described in this section.

Mapping inferred to reference strains: Given Demixer's output, we would like to know if any of the inferred strains output by Demixer can be mapped to a known reference strain (whose unique mutations/SNP-alleles are in a database called RDB1; see Suppl Section 1.2). For each inferred strain q $(1 \leq q \leq K)$, we check if it is the same as a particular reference strain p by first computing a dissimilarity measure (Kullback–Leibler or KL divergence D_{KL} (Cover 1999) of the inferred distribution $\boldsymbol{\phi}_q$ from the expected reference strain distribution $\boldsymbol{\phi}_p^{\text{ref}}$) as given in Equation 7 below. Let N_p be the set of SNPs such that one of its alleles uniquely identifies the reference strain p, i.e., $N_p = \{n : \left(\sum_{v \in \{A,C,G,T\}} \text{RDB1}[(n,v),p]\right) = 1\}$. Then,

$$D_{(KL)}(p \mid\mid q) := \frac{1}{|N_p|} \sum_{n \in N_p} D_{KL}(\boldsymbol{\phi}^{\text{ref}}_{p,n} \mid\mid \boldsymbol{\phi}_{q,n}) \tag{7}$$

where the Strain-SNP-allele distributions $\boldsymbol{\phi}^{\text{ref}}_{p,n}$ is given by the four-element vector $\text{RDB1}[(n,v),p]_{v \in \{A,C,G,T\}}$, and $\boldsymbol{\phi}_{q,n}$ is as estimated by Demixer.

The inferred strain q is assigned to the reference strain for which the KL divergence value is minimum and at most 1.5. If there are multiple such strains, we map q to the reference strain that is deepest in the hierarchical tree relating these strains (and finally if ties still remain, which may happen rarely, we break the ties arbitrarily). If the KL divergence is more than 1.5 for all reference strains, then strain q is called as a *de novo* or unmapped strain. Note that any inferred strain whose proportion is 0 in all the samples is excluded from all post-processing analyses.

Note that the above procedure maps each inferred strain to zero or one reference strain, but a reference strain could be mapped to 0, 1, or more inferred strains. The proportion of a reference strain mapping to more than one inferred strains is simply the sum of the estimated Sample-Strain proportions of the corresponding inferred strains.

Fine-tuning the mapped strains using the lineage tree: Complex datasets (like real-world datasets or simulated datasets mimicking real-world scenarios) may cause our Demixer to call a sample that contains only a single lineage (pure) as a mixture of different strains/sub-lineages within the single lineage (mixed infection). This may happen if additional mutations of the lineage missing from the reference database are present in the sample. To mitigate this issue, we perform sample-level post-processing for a dataset of interest using the lineage tree, which is a hierarchical tree relating all lineages/sub-lineages in the reference database (derived using naming conventions; e.g., 1.2.1 is a sub-lineage of 1.2). For each sample, we consider the set of reference strains mapped to at least one inferred strain in the sample, and process these reference strains one pair at a time in a certain fixed ordering. If a reference strain pair has an ancestor-descendant relation in the lineage tree, then we will retain only the tree node with the larger estimated proportion and add to it the smaller proportion of the other node. For instance, if the inferred strains of sample m gets mapped to reference lineages 1.2 (p) and 1.2.1 (p'), then we retain only lineage 1.2.1 if $\boldsymbol{\theta}_{m,p'} > \boldsymbol{\theta}_{m,p}$ and only 1.2 otherwise. After processing all such reference strain pairs, the set of retained strains along with the unmapped (*de novo*) inferred strains if any constitute the final strains called for the sample. A sample with only one final strain is reported as non-mixed/pure by Demixer (see Algorithm 2 in Supplementary Information for detailed steps of the post-processing heuristic). Please note that in the application of Demixer to all samples analyzed in this work, we detected either 0 or 1 *de novo* strains per sample; with the exception of a single sample (in the CRyPTIC dataset) where two *de novo* strains were detected – for simplicity, we merged both these strains into a single *de novo* strain by adding up their proportions as above.

Quality checks using SNP plots/modes and *de novo* filtering: Complex/noisy datasets also calls for the use of additional SNP-based quality checks and filters during post-processing. For each sample m in a dataset of interest, we plot the histogram of minor allele proportions of heterozygous SNPs. The x-axis of this histogram plot (also called the SNP plot) corresponds to the minor allele proportions of the heterozygous SNPs and the y-axis corresponds to the frequency (count) of such SNPs. Note that a SNP n is heterozygous in sample m if it has reads supporting at least two alleles, i.e., if $\sum_v \mathbb{1}_{\{\boldsymbol{S}_{m,n,v}>0\}} \geq 2$. Also, if a SNP has more than one minor allele, we sum up all these alleles' proportions to get the minor allele proportion for this plot. From the SNP or histogram plot of sample m, we can inspect the highest frequency histogram bin to estimate the mode of the minor allele proportions of heterozygous SNPs (denoted o_m; see Algorithm 2 for details) as well as its corresponding frequency (the number of SNPs, denoted f_m).

Given a dataset, we inspect the distribution of f_m values across all samples in the dataset, and use it to assign a confidence measure to each specific sample identified as mixed by Demixer. Let cutoff1 be the 25th and cutoff2 the 75th percentile of $\{f_m\}_{m=1}^M$; note that these cutoffs are expressed in terms of the number of SNPs. Then for a specific sample m, we apply the thresholds $f_m <$ cutoff1, cutoff1 $\leq f_m \leq$ cutoff2, and $f_m >$ cutoff2 to categorize Demixer's prediction for the sample as low, medium, and high confidence call respectively. Additionally, if Demixer's prediction for the sample (after the post-processing steps above) results in more than one inferred strains, with one mapped to a reference strain and the rest being *de novo* strain(s), we report the sample as mixed only if it's a high confidence call; otherwise, we remove (filter out) the *de novo* strain(s) and thereby report the sample as non-mixed with only the single reference strain.

These confidence cutoffs and *de novo* filtering will influence different downstream analyses. For $F1$ score calculation (see Suppl Section 1.5), the *de novo* filtering step will have an effect, especially for low-coverage benchmark datasets. For the Malawi dataset, we use these cutoffs to compare the performance of Demixer with an existing work MixInfect. For the CRyPTIC dataset, we apply similar thresholds ($f_m >$ cutoff2 and $o_m > 0.05$) to call a mixed sample detected by Demixer as high confidence, and thereafter test for association between drug resistance and mixed infection. Note that the f_m and o_m thresholds respectively ensure a sufficient number of SNPs and reads support the minority strain.

Hierarchical clustering of the inferred strains, and *in vitro* dataset processing: We compute the KL divergence measure between the inferred Strain-SNP-allele distribution of each strain q' from every other strain $q > q'$ using the Equation below, resulting in a lower-triangular KL matrix of size $K \times K$. This matrix is given as input to the hierarchical clustering algorithm (implemented using hclust function of R (R Core Team 2013) stats package) to obtain the tree structure of the inferred strains. The nodes in the tree captures the lineages/sub-lineages of the inferred strains.

$$D_{(KL)}(q \parallel q') := \frac{1}{N} \sum_{n=1}^{N} D_{KL}(\boldsymbol{\phi}_{q,n} \parallel \boldsymbol{\phi}_{q',n})$$

For the *in vitro* dataset, since the ground-truth is given in terms of majority and minority proportions, we aggregated the inferred strains in each sample into at most two strains by clustering the closer strains. Specifically, for each sample we clustered the strains using the above tree structure at different heights, starting from the leaves and until two clusters remain (using the cutree function of R). This procedure results in one clustered strain or two clustered strains called for each sample. The sum of the proportions of strains in each cluster is then taken as the majority/minority proportions present in the sample.

We perform two types of evaluation using these calls on the *in vitro* dataset. In proportion-based evaluation, the true and estimated proportions are plotted and compared with each other, regardless of the clustered strain identities (in order to facilitate comparison of Demixer with methods which doesn't infer strain identities like SplitStrains). In strain-identity-based evaluation, we inspect if the lineage of the clustered strains (i.e., the consensus lineage of all the inferred strains within the cluster) is the same as the ground-truth lineage. The lineage of an inferred strain is obtained as before (using the KL divergence based mapping procedure above, refined using the above clustering procedure instead of the fine-tuning procedure using the lineage tree).

1.4. Datasets used in the analysis

Various synthetic and real-world datasets have been used in our study to tune and evaluate Demixer. Certain synthetic datasets were used for hyperparameter tuning alone (and not for evaluation). The remaining synthetic or *in vitro* benchmarking datasets generated using different procedures were used for evaluations. These datasets, which help tune, understand and validate our Demixer method, are described below (see Suppl Table S1 for dataset sizes).

LDAmix1 and LDAmix2 datasets: These synthetic datasets were created based on the modified LDA's generative process used in the Demixer method as follows. Dataset LDAmix1 consists of 100 samples, and each sample is composed of 3 mixed strains. Each strain consists of 50 distinct SNPs. The proportion of the majority strain (strain 1) is drawn from the Gaussian distribution $\mathrm{N}(0.6, 0.05)$ with a mean 0.6 and standard deviation 0.05, that of the minority strain (strain 2) from another Gaussian $\mathrm{N}(0.2, 1)$ with a mean 0.2 and standard deviation 1, and the remaining proportion pertains to strain 3. LDAmix2 consists of 100 samples with 4 mixed strains, each with 50 unique SNPs. The minority strain (strain 4) is present only in 10% of the samples in order to determine the ability of Demixer to detect the strains present in a deficient proportion in fewer samples. The majority strain (strain 1) is drawn from $\mathrm{N}(0.6, 0.05)$, strain 2 from $\mathrm{N}(0.2, 1)$, strain 4 from $\mathrm{N}(0.1, 0.05)$, and strain 3 constitutes the remaining proportion. Each sample in both of these datasets consists of "n (no.of strains) $\times$ 50" SNPs with 100 reads for each SNP. Please note that we could also have used Dirichlet instead of Gaussian distributions for sampling the strain proportions above.

ART-TBmix1 and ART-TBmix2 datasets: The strains in ART-TBmix1 samples are chosen from 2,165 strains in QuantTB's (Anyansi et al. 2020) reference database, and that in ART-TBmix2 are chosen from 89 strains from Robust Barcoding database (Napier et al. 2020) (ignoring lineage 4 and 4.9 strains). The representative strains in both these datasets are generated by replacing the SNPs related to that strain in the reference H37Rv genome. The WGS reads for each sample are generated using the ART simulator (Version 2.5.8) with the default settings for the Illumina HiSeq 2500 platform, at a read length of 101 bp, per base sequence quality scores 20 to 30 and the quality scores are shifted by 9 to introduce sequencing errors as described in (Anyansi et al. 2020) and (Gabbasov et al. 2021). The coverage option is set accordingly for generating strains at different coverages 10x, 20, 70x, 30x, and 90x respectively for the different subsets. The four-strain sample is generated by selecting four strains at random from the respective databases and paired-end WGS reads for the individual strains are simulated at the specific coverage level. The reads of all four strains are then combined to generate the mixed sample. In the same manner, two strains are generated at two different coverages (for instance, 70x and 30x), to synthesize a sample mixed with two strains at 70x-30x coverage.

ARTmix dataset: ARTmix was generated to determine whether Demixer could delineate the strains accurately under different scenarios such as when all the references are known vs. only when a few references are known. As mentioned above, ARTmix dataset consists of 50 samples to emulate the real-world conditions where various samples exhibit diverse combinations of mixed strains. Each sample is either a single strain or a mixed strain composed of 2 or 3 strains, with the number of strains (1, 2, or 3) in a sample picked uniformly at random. Each strain in the sample is selected at random from a reference set of 7 strains. The reference strains are obtained by replacing nearly 100 SNPs in the reference H37Rv genome and the WGS data is generated as per the settings described in SplitStrains (Gabbasov et al. 2021) work using the ART simulator. In the case of two strain mixed samples, the majority strain (strain 1) is drawn from $\mathrm{N}(a, 1)$, such that $40 \leq a \leq 70$ is a random integer and strain 2 constitutes the remaining proportion. Similarly, for the three strain mixed samples, majority strain (strain 1) is drawn from $\mathrm{N}(a, 1)$, such that $60 \leq a \leq 70$ is a random integer, strain 2 is drawn from $\mathrm{N}(b, 1)$, such that $0 \leq b \leq 20$ is a random integer and strain 3 constitutes the remaining proportion.

***in vitro* dataset:** We observed a few discrepancies on examining the ground-truth information pertaining to *in vitro* dataset and have made changes to the lineages of the strains present in 8 mixed samples. Specifically, CAS1-Delhi is assigned its original lineage 3 (Conceição et al. 2023), and LAM11-ZWE is assigned lineage 4 (Mogashoa et al. 2019). The modified lineages and the lineages identified by Demixer and QuantTB are reported in Suppl File D1.

Malawi and CRyPTIC datasets: The Malawi TB isolates are collected from patients in Malawi, a Southeastern African region having higher rates of TB and HIV co-infection (Guerra-Assunção et al. 2015). Samples were collected from TB patients at regular intervals during the period 1996-2010 to understand whether the prevalence of HIV has any role in reinfection/relapse of TB. We have also analyzed 1963 isolates (downloaded from the publicly available datasets with accession numbers PRJEB2794 and PRJEB2358) as in (Sobkowiak et al. 2018). CRyPTIC is a collaborative effort that is focused on the better identification of drug-resistant TB by analyzing the WGS data. They work with TB research institutions from 27 countries worldwide and have collected WGS data of nearly 12289 isolates along with their responses to 13 tuberculosis drugs (CRyPTIC 2022a)(CRyPTIC 2022b). We used 12287 isolates that we were able to download in a proper vcf format in our analysis.

1.5. Evaluation metrics

The different metrics used to compare the performance of Demixer with other methods are as follows:

i) **Relative Error (RE):** We report relative error in simulated or *in vitro* datasets where we know the true/actual strain composition of a sample. The performance of a model/method on a given benchmark dataset is assessed by calculating the average relative error between the actual and predicted proportions for a strain k across all samples in that dataset (denoted RE_k), and further averaging RE_k across all strains $k \in \{1, ..., K\}$ to obtain the final relative error (RE). Note that this averaging over all K strains is possible, since we assume that our mapping of inferred to ground-truth strains (following the same procedure explained above for mapping inferred to reference strains) follows a one-to-one relation. For all the datasets and methods where relative error is computed, this assumption held true. Note that in a given dataset, the relative error RE_k of the kth strain can be computed as:

$$RE_k = \frac{1}{M} \sum_{m=1}^{M} \frac{(\mid \boldsymbol{\theta}_{m,k}^{\mathrm{GT}} - \boldsymbol{\theta}_{m,k} \mid)}{\boldsymbol{\theta}_{m,k}^{\mathrm{GT}} + 0.01}$$

where $\boldsymbol{\theta}_{m,k}^{\mathrm{GT}}$ and $\boldsymbol{\theta}_{m,k}$ are respectively the actual (ground-truth) and predicted (estimated) proportion of strain k in sample m. The pseudocount 0.01 (minimum proportion of 1%) is added to the denominator to account for zero values of $\boldsymbol{\theta}_{m,k}^{\mathrm{GT}}$.

ii) **F1 score:** QuantTB and Demixer are comparatively evaluated on benchmark datasets using the $F1$ score outlined in (Anyansi et al. 2020) as the metric. The $F1$ score of a sample quantifies how well a model/method can predict the actual strains present in the sample, which can then be averaged across all samples to get the $F1_{avg}$ score for a dataset. Before calculating the $F1$ scores, all post-processing steps above excepting the last one related to hierarchical clustering are followed as described, but using the ground-truth strains of a benchmark dataset as the set of reference strains (along with its associated lineage tree). For each sample, the outcome of these post-processing steps is a one-to-one relation between the inferred and ground-truth strains; using which we can define a true positive (inferred strain mapped to a corresponding ground-truth strain), a false positive (unmapped inferred strain), and a false negative (unmapped ground-truth strain). These can in turn be used to calculate the F1 scores for the sample.

1.6. Other aspects of Demixer

1.6.1. Comparison of Demixer and traditional LDA

One of the key differences between our proposed Demixer and traditional text LDA is that instead of having a single $\boldsymbol{\phi}$ matrix for all the SNP-allele combinations, we have a $\boldsymbol{\phi}$ matrix for each SNP. To empirically understand this difference, we applied traditional LDA and Demixer in *de novo* mode (no reference database given) on a special dataset where samples have strains mixed in roughly equal proportions. Note that the inference of mixed strains' identities/proportions in this dataset is difficult due to the equal proportions of strains. This dataset specifically comprised 53 samples (2 pure and 51 mixed with 2 synthetic strains) such that the proportion of the majority strain ranges from 49 to 100% in 1% increment in the mixed samples. Each synthetic strain is obtained by replacing 100 random SNPs in the reference H37Rv genome. We used Python Gensim library for the traditional LDA modeling (Řehůřek and Sojka 2010). In this implementation, each read that supports a SNP-allele combination is represented as a word in a document, and then fed to the LDA model. The traditional LDA neither directly captures the sequencing depth (i.e., the number of reads mapping to a SNP) nor the distribution of alleles within each SNP, whereas this aspect is inherently built within the framework of our SNP-LDA model. The proportion of one of the strains determined by traditional text LDA and Demixer on the samples in this dataset was compared with its actual proportion. Traditional LDA was unable to determine the proportions accurately for the samples with equal proportions of strains. The ratios determined by Demixer were very close to the actual ratios, as illustrated in Suppl Figure S4, indicating that our proposed modeling approach determines the proportions with high accuracy.

1.6.2. Model selection and hyperparameter tuning

Symmetric or asymmetric priors can be chosen for the hyperparameters α and β of the LDA model. In symmetric prior, the assumption is that each strain is equally likely in a sample and each SNP-allele is equally likely in a strain, whereas asymmetric prior allows certain strain/SNP-alleles to occur more often than others in a set of samples. Though symmetric priors are commonly chosen, Wallach et al. (Wallach et al. 2009) have shown the advantages of choosing asymmetric Dirichlet prior over the document-topic distribution, whereas there is limited improvement in choosing asymmetric prior over word-topic distribution. A similar hypothesis has been put forward in (Stevens et al. 2012) by testing different combinations of symmetric and asymmetric priors over $\boldsymbol{\phi}$ and $\boldsymbol{\theta}$ distributions using coherence score and interpretability measures. In (Petterson et al. 2010), topic-dependent smoothing coefficients for words were employed to facilitate the identification of topics with incoherent words, rather than relying on an asymmetric prior for topic-word distribution. We follow a similar method here.

Specifically, we employed 5 different hyperparameter combinations $(1, 0.01)$, $(0.01, A)$, $(0.01, B)$, $(A, 0.01)$ and $(B, 0.01)$ (including asymmetric prior for α parameter and topic smoothing coefficients for β parameter) to identify the default hyperparameters for the model. The first element in each set corresponds to the α hyperparameter and the second element to the β hyperparameter. $(1, 0.01)$ is the simple prior chosen assuming that a sample contains only a few minority strains and either the reference or alternate allele occurs with higher probability at a SNP position. For the asymmetric combination, if the number of strains is assumed to be 3, A will take the values [0.01, 1, 1] and B [0.01, 10, 1]. Here, 0.01 indicates the reference strain for which the SNP-alleles are known, and the remaining values correspond to the *de novo* strains. A is assigned values assuming that the minority strains are equally likely to occur, whereas, in B, the assumption is that among the minority strains, one would occur in the majority than the other. Similarly, for 4 strain cases, A and B would correspond to [0.01, 1, 1, 1] and [0.01, 10, 1, 1] respectively.

1.6.3. Comparison of Demixer with embedding-based methods

We chose LDA over popular word-embedding-based deep learning (DL) models to develop Demixer, due to LDA being a bag-of-words model and therefore more suited to identify different strains in a sample and estimate their proportions (by viewing each strain as a bag of mutations). Extensive literature survey did not reveal any word-embedding-based DL models that directly address the mixed infection detection problem. However, we were able to find DL models (e.g., DeepMicrobes (Liang et al. 2020) and DNABERT-2 (Zhou et al. 2023)) that convert microbial DNA sequences to numerical vectors (embeddings) and use such embeddings to improve the taxonomic classification of short metagenomic reads. To compare Demixer with DeepMicrobes/DNABERT-2, we adapted these models as described below to perform (i) strain/lineage-level instead of genus/species-level classification of reads, and (ii) aggregate the class prediction of reads in a sample to predict the majority strain in the sample. As such, this is not a direct one-to-one comparison due to the adaptation step, however this is the best we could do to assess Demixer alongside certain popular DL models.

We performed two types of comparisons of Demixer with the adapted DL models: (i) read-level and (ii) sample-level, as explained next.

Read-level comparison: To perform this comparison, we needed a separate read-level dataset (distinct from the sample-level benchmark datasets used in this study) to adapt/train the DeepMicrobes and DNABERT-2 models. Towards this end, we first selected 10 reference strains belonging to TB lineages 1, 2 and 3 from Demixer's reference database. These strains/lineages were selected (and others such as lineage 4 excluded) to obtain a balanced dataset of reads that would cover a similar number of lineage-specific mutations (also known as lineage-defining mutations, since each such mutation is present exclusively in a lineage). Next, we used ART simulator to derive the actual set of reads for each selected strain as follows: replace the H37Rv genomic sequence with the strain-specific mutations, sample paired-end

artificial reads from this strain-specific genome using ART simulator, and keep only those reads covering the strain-specific mutations. This resulted in a total of 35665 reads spread across the 10 selected strains belonging to the three TB lineages, with each read covering at least one lineage-defining mutation.

The read-level dataset generated above can now be used to adapt DeepMicrobes/DNABERT-2 as follows:

- We retrained the DeepMicrobes model using the read-level dataset according to the instructions given by DeepMicrobes authors (https://github.com/MicrobeLab/DeepMicrobes/), except for changing the confidence threshold from the default 50% value to 90% to improve its performance.
- DNABERT-2 model, which was originally trained using the genomes of multiple species, was fine-tuned using the same read-level dataset. After fine-tuning, the resulting embeddings were used to train a Logistic Regression (LR) model to classify reads of new samples (note that changing the LR model to alternate models such as Support Vector Machines (SVM) and eXtreme Gradient Boosting (XGBoost) yielded comparable results). While fine-tuning, we set most hyperparameters to the default values suggested by the DNABERT-2 authors, with the following exceptions: maximum read length set to 50, batch size of 16, learning rate at 2×10^{-5}, 5 training epochs, 100 warm-up steps, 95/5 training-validation split and inverse square root scheduler (https://github.com/MAGICS-LAB/DNABERT_2). These exceptions were made because the default parameter values of DNABERT-2 model did not perform well in the test dataset.

To perform a preliminary assessment of the embeddings learnt by DeepMicrobes, DNABERT-2, and Demixer, we simulated a new set of 300 reads containing lineage-defining mutations (from TB lineages 1, 2 and 3 as mentioned above). For Demixer, we considered the probability distribution of strains for each SNP-allele (mutation) as its corresponding embedding, with this probability distribution given by the $\boldsymbol{\phi}$ matrix of the Demixer model trained on the CRyPTIC dataset. Upon visualizing the embeddings from the three methods using a t-distributed Stochastic Neighbor Embedding (tSNE) plot, we observe that all the three models cluster the reads according to their lineage (see Suppl Figure S9). This encouraged us to further evaluate the classification performance of the three models.

Sample-level comparison: To assess the sample-level classification performance of different methods, we used the *in vitro* dataset consisting of samples belonging to lineages 1, 2, 3, and 4. To facilitate comparison between our semi-supervised Demixer model and the supervised models DeepMicrobes and DNABERT-2, we proceeded as follows.

- Demixer was trained on the *in vitro* dataset after hiding the SNP-alleles (mutations) of lineage 4 and by keeping only the SNP-alleles of lineages 1, 2 and 3 in the reference database. The model parameter K is set to 5 (as per $K'+2$ heuristic). Note that Demixer successfully delineated lineage 4 samples, even in the absence of prior information for this lineage.
- We started with the DeepMicrobes and DNABERT-2 models trained and fine-tuned respectively using the read-level dataset comprising lineages 1, 2 and 3 discussed above (so the SNP-alleles of lineage 4 are hidden here too), and aggregated their read-level predictions to make sample-level estimations as below.
  - Being supervised models, DeepMicrobes and DNABERT-2 models can assign the reads in a sample to only the known lineages 1, 2 or 3. For a given sample, we estimated its lineage proportions simply using the ratio of the number of reads assigned to a particular lineage to the total number of reads in the sample.
  - Given the above estimated proportions, the majority lineage of a sample is taken to be the class/lineage with the highest proportion, but only if it exceeds a certain threshold (0.5 for DeepMicrobes and 0.37 for DNABERT-2, because the default value of 1/(# of classes) = 1/3 or other values we tested gave poor performance for these models). If the threshold is not exceeded, the majority lineage of the sample is assumed to be the novel lineage 4.

Upon evaluating the above three methods on the *in vitro* dataset with pure and mixed samples, we observed that all three methods perform comparably in identifying the majority strain, with Demixer demonstrating similar or higher accuracy (see Suppl Table S8). DeepMicrobes and DNABERT-2 were also able to delineate the majority strain identities, but they faced challenges in accurately estimating the corresponding proportions (see Suppl Figure S10). Demixer's estimation of majority strain proportion is closer to the actual proportion than that of DeepMicrobes and DNABERT-2.

Finally, Demixer can also learn the mutational profiles of both known and novel lineages/strains as part of its multi-sample inference procedure, unlike embedding-based models which cannot yield such profiles. For instance, in this analysis, Demixer had no prior information on lineage 4 mutations, yet the analysis of mutation profile ($\boldsymbol{\phi}$ parameter) determined by Demixer revealed mutations of lineage 4 (specifically sub-lineages 4.9 and 4.3.4.2.1) in the novel strains. As for improving the performance of embedding-based methods in delineating strain proportions and identities, including that of novel strains, further refinements would be needed to handle non-lineage-defining mutations and novel lineages. Furthermore, the complexity of training embedding-based models will increase with the inclusion of all sub-lineages (instead of just the three higher-level lineages considered in the current comparison).

1.6.4. Implementation details

All the experiments are conducted on an Intel(R) Xeon(R) Platinum 8180 CPU @ 2.50GHz processor running CentOS with 112 cores and 1 TB RAM. The code for preprocessing the input vcf file is implemented in Python. The tools from Python *scikit-allel* framework are used for reading and processing the vcf files. The parallelization of the CGS algorithm is implemented using the OpenMP library. The gsl_ran_multinomial function from GNU Scientific Library (GSL) is utilized to generate random samples from a multinomial distribution in the CGS algorithm. The postprocessing steps of Demixer are implemented again in Python using the estimated model parameters of the SNP-LDA model. For simplicity, we have reduced the 3D matrices $\boldsymbol{S}$ and $\boldsymbol{\phi}$ to 2D during the code development of Demixer. We used the programming language and statistical software environment R to perform hierarchical clustering of the strains detected in the *in vitro* dataset samples.

1.6.5. Using Demixer for analysing new sample(s)

Instead of learning Demixer separately on each dataset, we can also run Demixer in a training+testing mode, wherein the Demixer model learnt from a large diverse training dataset (CRyPTIC in our case) can be used to inspect the strains in a small test dataset containing one or more new samples. To facilitate this mode, Demixer is applied on the training dataset in an unsupervised (without class labels) fashion to learn the model parameters $\boldsymbol{\theta}^{\text{train}}$ and $\boldsymbol{\phi}^{\text{train}}$; of these, we utilize only the $\boldsymbol{\phi}^{\text{train}}$ parameter to learn the test samples' strain proportions $\boldsymbol{\theta}^{\text{test}}$. The test dataset goes through the same three steps of Demixer, preprocessing, estimating $\boldsymbol{\theta}^{\text{test}}$, and post-processing, but with a few key changes. In preprocessing, we ensure the same set of SNPs are analyzed in both training and test datasets (by using the force call option in FreeBayes tool with the merged multisample .vcf file of the training data as its input). When estimating $\boldsymbol{\theta}^{\text{test}}$, we initialize the strains of reads using the critical information in $\boldsymbol{\phi}^{\text{train}}$ parameter as shown in Equation 8 below, and run the rest of the hybrid SNP-LDA parallelized CGS algorithm as is for a fixed number of iterations (100 for single sample and 500 for a batch of samples to update $\boldsymbol{\theta}^{\text{test}}$ and $\boldsymbol{\phi}^{\text{test}}$, and eventually report the former and ignore the latter). Note that the CGS update formula (Equation 5) is used as is for the CGS iterations, but its revised form (Equation 8 below that uses $\boldsymbol{\phi}^{\text{train}}$ and assumes $\boldsymbol{\theta}$ is based only on its prior) is used for initialization alone.

$$\mathbb{P}(z_{m,n,r} = k \mid w_{m,n,r} = v, \boldsymbol{\phi} = \boldsymbol{\phi}^{\text{train}}, \boldsymbol{\theta} = \{\alpha_k\}) \propto \alpha_k \cdot \frac{(\boldsymbol{wt}_{n,v}\boldsymbol{\phi}^{\text{train}}_{k,n,v} + \beta_{n,v})}{(\sum_{v'} \boldsymbol{wt}_{n,v'}\boldsymbol{\phi}^{\text{train}}_{k,n,v'} + \beta_{n,v'})} \tag{8}$$

For this training+testing mode to work, the training dataset should be reasonably large and diverse. Specifically, a diverse set of strains (established reference strains as well as newly discovered strains) should be represented in a sufficiently large number of samples in the training dataset. Large training datasets with sufficient sequencing depth can also allow us to mitigate errors if any in the mutations recorded in the reference database. Based on these factors and our analysis of applying Demixer on different datasets of sizes ranging from 48 to 12,287, we propose a heuristic that the training dataset must have at least 100 samples when learning a Demixer model in the training+testing mode.

References

Andrews S et al. FastQC: a quality control tool for high throughput sequence data, 2017.

Anyansi C, Keo A, Walker BJ et al. QuantTB – a method to classify mixed Mycobacterium tuberculosis infections within whole genome sequencing data. *BMC Genomics*, 21(1):1–16, 2020.

Blei DM, Ng AY, Jordan MI. Latent Dirichlet Allocation. *Journal of Machine Learning Research*, 3(Jan):993–1022, 2003.

Carpenter Bob. Integrating out multinomial parameters in Latent Dirichlet Allocation and Naive Bayes for Collapsed Gibbs Sampling. *Rapport Technique*, 4:464, 2010.

Chen J, Li K, Zhu J, Chen W. WarpLDA: a Cache Efficient O(1) Algorithm for Latent Dirichlet Allocation. *Proceedings of the VLDB Endowment*, 9 (10), 2016.

Conceição EC, da Conceição ML, Marcon DJ et al. Genomic Diversity of the rarely observed genotype of the Mycobacterium tuberculosis Central Asian (CAS) Lineage 3 from North Brazil. *Microorganisms*, 11(1):132, 2023.

Cover TM. *Elements of information theory*. John Wiley & Sons, 1999.

CRyPTIC Consortium. A data compendium associating the genomes of 12,289 Mycobacterium tuberculosis isolates with quantitative resistance phenotypes to 13 antibiotics. *PLoS Biology*, 20(8):e3001721, 2022a.

CRyPTIC Consortium. Genome-wide association studies of global Mycobacterium tuberculosis resistance to 13 antimicrobials in 10,228 genomes identify new resistance mechanisms. *PLoS Biology*, 20(8):e3001755, 2022b.

Danecek P, Bonfield JK, Liddle J et al. Twelve years of SAMtools and BCFtools. *Gigascience*, 10(2):giab008, 2021.

Davis CS. The computer generation of multinomial random variates. *Computational statistics & data analysis*, 16(2):205–217, 1993.

Gabbasov E, Moreno-Molina M, Comas I et al. SplitStrains, a tool to identify and separate mixed Mycobacterium tuberculosis infections from WGS data. *medRxiv*, 2021.

Gordon SV, Parish T. Microbe Profile: Mycobacterium tuberculosis: Humanity's deadly microbial foe. *Microbiology*, 164(4):437–439, 2018.

Guerra-Assunção JA, Houben RMG, Crampin AC et al. Recurrence due to relapse or reinfection with Mycobacterium tuberculosis: a whole-genome sequencing approach in a large, population-based cohort with a high HIV infection prevalence and active follow-up. *The Journal of Infectious Diseases*, 211(7):1154–1163, 2015.

Jagarlamudi J, Daumé III H, Udupa R. Incorporating lexical priors into topic models. In *Proceedings of the 13th Conference of the European Chapter of the Association for Computational Linguistics*, pages 204–213, 2012.

Li H, Durbin R. Fast and accurate short read alignment with Burrows–Wheeler transform. *Bioinformatics*, 25(14):1754–1760, 2009.

Liang Q, Paul WB, Liu Y et al. DeepMicrobes: taxonomic classification for metagenomics with deep learning. *NAR Genomics and Bioinformatics*, 2 (1):lqaa009, 2020.

Liu L, Tang L, Dong W et al. An overview of topic modeling and its current applications in bioinformatics. *SpringerPlus*, 5(1):1–22, 2016.

Mogashoa T, Melamu P, Ley SD et al. Genetic diversity of Mycobacterium tuberculosis strains circulating in Botswana. *PLoS One*, 14(5):e0216306, 2019.

Napier G, Campino S, Merid Y et al. Robust barcoding and identification of Mycobacterium tuberculosis lineages for epidemiological and clinical studies. *Genome Medicine*, 12(1):1–10, 2020.

Pedregosa F, Varoquaux G, Gramfort A et al. Scikit-learn: Machine Learning in Python. *Journal of Machine Learning Research*, 12:2825–2830, 2011.

Petterson J, Buntine W, Narayanamurthy S et al. Word Features for Latent Dirichlet Allocation. *Advances in Neural Information Processing Systems*, 23, 2010.

R Core Team. *R: A language and environment for statistical computing*, 2013.

Řehůřek R, Sojka P. Software framework for topic modelling with large corpora. In *Proceedings of the LREC 2010 Workshop on New Challenges for NLP Frameworks*, pages 45–50, 2010.

Sobkowiak B, Glynn JR, Houben RMGJ et al. Identifying mixed Mycobacterium tuberculosis infections from whole genome sequence data. *BMC Genomics*, 19(1):1–15, 2018.

Stevens K, Kegelmeyer P, Andrzejewski D, Buttler D. Exploring topic coherence over many models and many topics. In *Proceedings of the 2012 Joint Conference on Empirical Methods in Natural Language Processing and Computational Natural Language Learning*, pages 952–961, 2012.

Wallach H, Mimno D, McCallum A. Rethinking LDA: Why priors matter. *Advances in Neural Information Processing Systems*, 22, 2009.

Wilson AT, Chew PA. Term weighting schemes for Latent Dirichlet Allocation. In *Human Language Technologies: The 2010 annual conference of the North American Chapter of the Association for Computational Linguistics*, pages 465–473, 2010.

Wood J, Tan P, Wang W, Arnold C. Source-LDA: Enhancing probabilistic topic models using prior knowledge sources. In *2017 IEEE 33rd International Conference on Data Engineering (ICDE)*, pages 411–422. IEEE, 2017.

Zhao W, Chen JJ, Perkins R et al. A novel procedure on next generation sequencing data analysis using text mining algorithm. *BMC Bioinformatics*, 17(1):1–15, 2016.

Zhou Z, Ji Y, Li W et al. DNABERT-2: Efficient foundation model and benchmark for multi-species genome. *arXiv preprint arXiv:2306.15006*, 2023.

2. Supplementary Algorithms

Algorithm 1 Parallel Collapsed Gibbs Sampling: This pseudocode pertains to Demixer's default mode known as hybrid SNP-LDA (to obtain non-hybrid SNP-LDA variants, the dictionaries $\mathrm{SD}_{m=1}^{M}$ or GD (see Suppl Section 1.2 for details of SD and GD) that map known SNP-alleles to the strains they are present should be emptied out before being provided as input). The algorithm begins by assigning strains to each read of a sample either at random or using reference SNP-alleles. During each CGS iteration, strains to each read of a sample are reassigned based on $\boldsymbol{\theta}$ and $\boldsymbol{\phi}$, followed by the updation of these matrices.

Input: Sample-SNP-allele matrix $\boldsymbol{S}_{M\times N\times 4}$, SNP weights $\boldsymbol{wt}_{N\times 4}$, the sample-specific dictionaries $\mathrm{SD}_{m=1}^{M}$, and global dictionary GD (with $SD_m[j]$ or $GD[j]$ mapping SNP-allele j to the set of strains present in the m-th sample or across all samples respectively).

Output: Inferred model parameters – Sample-Strain matrix $\boldsymbol{\theta}_{M\times K}$, Strain-SNP-allele matrix $\boldsymbol{\phi}_{K\times N\times 4}$.

```
K ← K' + 2        (determined as per the heuristic for choosing K)
Initialize θ, φ, C_{K×M×N×4} to zeros
# pragma omp parallel for
for i = 1, 2, ..., M do
    for j = 1, 2, ..., N do
        for v = 1, ..., 4 do
            if (j, v) in SD_i then
                u ← prob(SD_i[(j, v)])
            else
                u ← [1/K, 1/K, ..., 1/K] (K-length vector)
            end if
            C_{1:K,i,j,v} ← Multinomial(S_{ijv}, u)
            Update θ_{i,1...K} using C_{1:K,i,*,*}
        end for
    end for
end for
Update φ using C
for iter = 1, 2, ..., max_iterations do
    #pragma omp parallel for
    for i = 1, 2, ..., M do
        for j = 1, 2, ..., N do
            a_{i,1:K} ← θ_{i,1:K}
            b_{1:K,j,1:4} ← wt_{j,1:4} * φ_{1:K,j,1:4}
            θ_{i,1:K} ← θ_{i,1:K} − (wt_{j,1:4} * C_{1:K,i,j,1:4})
            for v = 1, ..., 4 do
                if (j, v) in GD then
                    strains ← GD[(j, v)]
                    u ← a[strains] .* b[strains]
                else
                    u ← a_{1:K} .* b_{1:K}
                end if
                C_{1:K,i,j,v} ← Multinomial(S_{ijv},u)
                Update θ_{i,1:K} using C_{1:K,i,*,*}
            end for
        end for
    end for
    Update φ using C
end for
for m = 1, 2, ..., M do
    for k = 1, 2, ..., K do
        θ_{m,k} = C_{k,m,*,*} / C_{*,m,*,*}
    end for
end for
for k = 1, 2, ..., K do
    for n = 1, 2, ..., N do
        for v = 1, ..., 4 do
            φ_{k,n,v} = C_{k,*,n,v} / C_{k,*,n,*}
        end for
    end for
end for
```

- prob($\mathrm{SD}_i[(j, v)]$): If $\mathrm{SD}_i[(j, v)]$ returns more than 1 strain, then the strain at a deeper level in the hierarchy will be assigned a higher probability (0.95) than the other strains. In case of ties, the strain that is seen first during processing will be assigned higher probability.
- .* indicates element-wise multiplication.

Algorithm 2 Post-processing steps of Demixer: A brief overview of the postprocessing steps and the order in which the different postprocessing steps are done by Demixer is shown here. Please refer Suppl Section 1.3.3 for a detailed description of each postprocessing step.

Input: Inferred model parameters, $\boldsymbol{\theta}$, $\boldsymbol{\phi}$, output by Algorithm 1

Output: Phylogeny to visualize the inferred strains; Final Proportions $\boldsymbol{FP}_{M\times L}$, Final Strains $\boldsymbol{FS}_{M\times L}$, and (Mixed/Non-mixed) Assignments $\boldsymbol{AS}_M$

Note: L indicates the maximum number of strains that could be present in a sample after postprocessing/merging. Also, $\boldsymbol{FP}_{M\times L}$ stores the final refined proportions, $\boldsymbol{FS}_{M\times L}$ the final strains, and $\boldsymbol{AS}_M$ the mixed vs. non-mixed assignment for each of the M samples (after postprocessing/merging).

/* **Hierarchical clustering of the inferred strains:** */
Compute and output a phylogeny relating all the inferred strains, excepting strains whose estimated proportion is zero across all samples (R hclust function is used as described in Suppl Section 1.3.3).

/* **Mapping inferred to reference strains:** */
Setup mapped_reference_strainid$_K$ array as follows. Map each inferred strain k to the closest reference strain p with KL-divergence at most 1.5 (as described in Suppl Section 1.3.3), and store this information as "mapped_reference_strainid[k] = p". If no such reference strain exists, then store "mapped_reference_strainid[k] = concat('*de novo* strain',k) ".

for $i = 1, 2, \ldots, M$ **do**
    prop_list ← non-zero-values($\boldsymbol{\theta}_m$) (get the non-zero strain proportions)
    id ← mapped_reference_strainid[non-zero-indices($\boldsymbol{\theta}_m$)] (get the id's of mapped reference strains with non-zero proportion)
    id ← sort(id); prop_list also sorted using same ordering; (sorting required for prefix check below)
    if len(id) = 1 **then**
        $\boldsymbol{AS}_m$ ← "Non-mix"; $\boldsymbol{FS}_m$ ← id; $\boldsymbol{FP}_m$ ← prop_list;
        continue to next sample
    end if

    /* **Fine-tuning (aka merging) the mapped strains using the lineage tree:** */
    for $j = 1, 2, \ldots, len(\text{id}) - 1$ **do**
        /* Prefix condition checks if strain j (e.g., lineage 4.2) is ancestor of strain j+1 (e.g., lineage 4.2.1) in the lineage tree */
        if id$_j$ = prefix(id$_{j+1}$) and ids $j, j+1$ are not *de novo* strains **then**
            if prop_list$_j$ > prop_list$_{j+1}$ **then**
                id$_{j+1}$ ← id$_j$ (Assign the id of the ancestor to the merged strain)
            end if
            prop_list$_{j+1}$ ← prop_list$_j$ + prop_list$_{j+1}$ (Merge)
            Flag strain j as "removed".
        else
            Do not merge
        end if
    end for
    $\boldsymbol{FS}_m$ ← ids of all strains NOT flagged as "removed" above; $\boldsymbol{FP}_m$ ← corresponding entries of prop_list;

    /* Special case handling: **Quality checks using SNP plots/modes and *de novo* filtering:** */
    prop$_m$ ← $[ma_1, ma_2, \ldots, ma_H]$ (ma_h is the minor allele proportion at h-th heterozygous SNP out of all H heterozygous SNPs in the sample)
    f_m ← frequency of the most-frequent bin in histogram(prop$_m$)
    o_m ← mode of prop$_m$ (estimated using the mode of ma_h values falling within the most-frequent histogram bin)
    /* Note: "$f_m >$ cutoff2" is a high confidence call; see Suppl Section 1.3.3 for details */
    if $\boldsymbol{FS}_m$ contains one reference strain and at least one *de novo* strain AND $f_m \leq$ cutoff2 **then**
        Remove (filter out) all *de novo* strain(s) from $\boldsymbol{FS}_m$, and update $\boldsymbol{FP}_m$ accordingly
    end if

    if len($\boldsymbol{FS}_m$) = 1 **then**
        $\boldsymbol{AS}_m$ ← "Non-mix"
    else
        $\boldsymbol{AS}_m$ ← "Mix"
    end if
end for

3. Supplementary Tables

Table S1. Summary of the datasets used in the study

Dataset	Number of Strains	Synthetic/ Real World	Number of Samples
LDAmix1	3	Synthetic - Demixer's Generative Process	100 (all mixed)
LDAmix2	4	Synthetic - Demixer's Generative Process	100 (all mixed)
ART-TBmix1	2,4	Synthetic - Art Simulator	800 (all mixed)
ART-TBmix2	2,4	Synthetic - Art Simulator	800 (all mixed)
ARTmix	1,2,3	Synthetic - Art Simulator	50 (18 pure & 32 mixed)
Malawi	unknown	Real-world Dataset	1963
CRyPTIC	unknown	Real-world Dataset	12287

Table S2. Empirical estimation of the constant D in Gibbs sampler's weighted update equation: Demixer is run on the *in vitro* dataset with different values for D ranging from 1 to 10 (100 runs for each value of D) to determine its optimal value in the update Equation 6 (and the related $\boldsymbol{wt}_{n,v}$ formula) in Suppl Section 1.3.2. The reported values are the average relative errors and standard deviations computed across 100 runs for each value of D.

D	0 (no weight)	1	2	3	4	5	6	7	8	9	10
**Weight ($\boldsymbol{wt}_{n,v}$)**	1	2	2	3	4	4	5	6	7	7	8
**Average Relative Error**	0.5432	0.5166	0.5589	**0.2920**	0.3413	0.3407	0.3866	0.4114	0.4282	0.4169	0.3897
**Standard deviation**	0.2157	0.2092	0.1950	**0.0052**	0.0216	0.0220	0.0449	0.0918	0.1369	0.1445	0.1860

Table S3. Weights estimated for different datasets: The weight assigned to the reference alleles in each of the synthetic and real-world datasets determined as per the $\boldsymbol{wt}_{n,v}$ formula described in Suppl Section 1.3.2 is shown. Recall that N', A, K, and D used in the formula represent the number of SNPs, the average number of reference SNP-alleles per sample, the number of strains, and the constant respectively.

Dataset	Type	N'	A	K	D	$\boldsymbol{wt}_{n,v}$	adjusted $\boldsymbol{wt}_{n,v}$
**LDAmix1 (synthetic)**	**non-hybrid**	150	0	3	3	0	1
	**hybrid**	150	50	3	3	2	2
**LDAmix2 (synthetic)**	**non-hybrid**	200	0	4	3	0	1
	**hybrid**	200	50	4	3	3	3
**ART-TBmix1 (synthetic)**	**10x**	359372	2203	786	3	1	2
	**20x**	95319	2321	672	3	1	2
	**70x-30x**	60034	1434	365	3	1	2
	**90x-10x**	59081	1396	365	3	1	2
**ART-TBmix1 (synthetic)**	**10x**	281228	439	83	3	24	24
	**20x**	6676	447	82	3	1	2
	**70x-30x**	5543	233	82	3	1	2
	**90x-10x**	5539	230	82	3	1	2
**ARTmix (synthetic)**	**all references known**	729	164	7	3	1	2
	***de novo* mode**	729	111	7	3	3	3
in vitro	**benchmark**	4818	313	19	3	3	3
**Malawi**	**real-world**	15625	246	43	3	5	5
**CRyPTIC**	**real-world**	74495	131	71	3	24	24

Table S4. Precision, Recall and $F1$ scores of Demixer and QuantTB on ART-TBmix1 dataset

Coverage	Demixer			QuantTB		
	Precision	Recall	$F1$ score	Precision	Recall	$F1$ score
**10x**	0.91	0.91	0.91	0.98	1.00	0.99
**20x**	1.00	0.99	0.99	0.98	1.00	0.99
**90x-10x**	0.99	0.99	0.99	0.99	1.00	0.99
**70x-30x**	1.00	1.00	1.00	0.99	1.00	1.00

Table S5. Precision, Recall and $F1$ scores of Demixer and QuantTB on ART-TBmix2 dataset

Dataset	Demixer			QuantTB		
	Precision	Recall	$F1$ score	Precision	Recall	$F1$ score
**10x**	0.95	0.88	0.91	1.00	0.79	0.87
**20x**	0.97	0.91	0.93	1.00	0.81	0.88
**90x-10x**	0.96	0.96	0.95	1.00	0.82	0.88
**70x-30x**	0.95	0.96	0.95	1.00	0.82	0.88

Table S6. Ablation analysis on *in vitro* dataset: Demixer is run on the *in vitro* dataset for the six different heuristic combinations (10 runs for each combination) to determine the best heuristic combination. The reported values are the average relative errors and standard deviations computed across 10 runs for each combination. Here, K is set to 30 (a high value) for the combinations involving $\overline{K}$ and 19 (based on $K' + 2$ heuristic) for the combinations involving K.

**Heuristic combinations**	$(\overline{R}, \overline{K}, \overline{W})$	$(\overline{R}, K, \overline{W})$	$(R, \overline{K}, \overline{W})$	$(R, \overline{K}, W)$	$(R, K, \overline{W})$	(R, K, W)
**Average Relative Error**	1.9274	0.9047	0.4858	0.4695	0.5168	**0.2931**
**Standard deviation**	0.6506	0.3448	0.2431	0.2396	0.2317	**0.0049**

Table S7. Time and space usage of Demixer for the experimental/real-world datasets: The time and space used by Demixer for preprocessing and running parallel CGS algorithm for 1000 iterations is shown. The memory usage reported here is the maximum virtual memory utilized as determined by the Unix top command.

Dataset	Run-time		Memory	
	Pre-processing	Parallel CGS	Pre-processing	Parallel CGS
in vitro	11.4 seconds	54 seconds	0.16 GB	4.067 GB
Malawi	3.57 minutes	20.41 minutes	1.61 GB	14.3 GB
CRyPTIC	1.62 hours	17.68 hours	46.51 GB	368.1 GB

Table S8. Performance comparison of DeepMicrobes, DNABERT-2, and Demixer in identifying the majority strain in the *in vitro* dataset: The effectiveness of the different methods in identifying the majority strain is assessed for three distinct cases: (1) predictions for samples with majority strain corresponding to lineages seen during training (known lineages), (2) predictions for samples with lineage 4 as the majority strain (novel lineage), and (3) predictions for all the samples (all lineages). For the analysis of only known lineages and all lineages, the metrics Precision, Recall and $F1$ score (calculated using functions from Python's scikit-learn library (Pedregosa et al. 2011)) are averaged using macro approach, whereas micro approach is used for the novel lineage case.

Method / Metric	Known lineages (1, 2 and 3)			Novel lineage (4)			All lineages		
	DeepMicrobes	DNABERT-2	Demixer	DeepMicrobes	DNABERT-2	Demixer	DeepMicrobes	DNABERT-2	Demixer
Accuracy	0.97	0.94	1	0.88	0.94	0.94	0.94	0.94	0.98
Precision	0.97	0.75	1	0.88	0.94	0.94	0.93	0.94	0.97
Recall	0.96	0.7	1	0.88	0.94	0.94	0.94	0.93	0.98
$F1$ score	0.96	0.72	1	0.88	0.94	0.94	0.93	0.94	0.98

4. Supplementary Figures

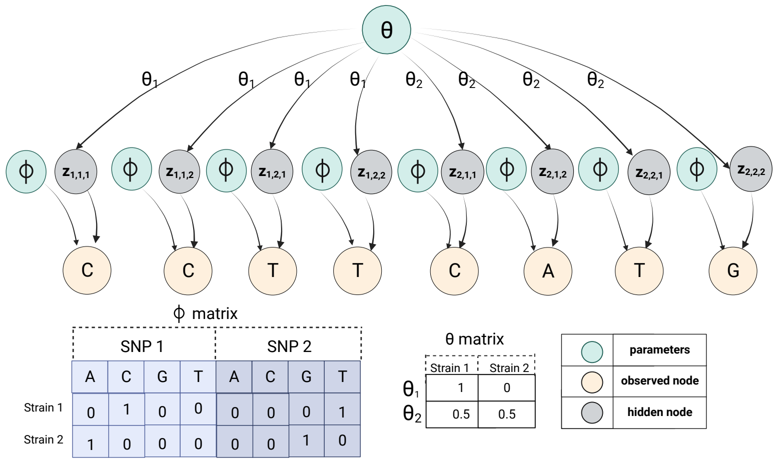

Fig. S1. Generative process of SNP-LDA: The assignment of strains to reads in a sample set consisting of 2 samples is shown. Each sample is assumed to have 2 SNPs with 2 reads per SNP. $\boldsymbol{\theta}$ and $\boldsymbol{\phi}$ are the model parameters and are assumed to be known.

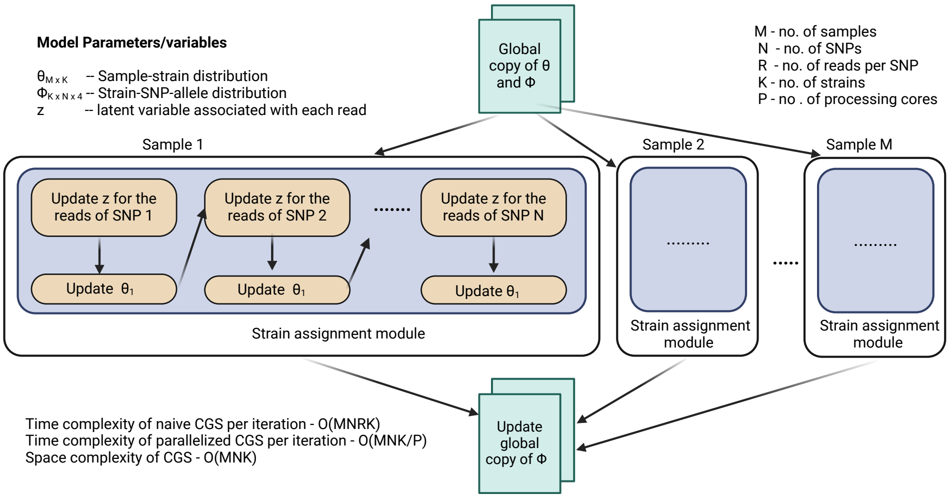

Fig. S2. Heuristics for parallelizing the CGS algorithm: In one CGS iteration, all the samples are processed in parallel by maintaining local copies of the $\boldsymbol{\phi}$ matrix. Each row in the $\boldsymbol{\theta}$ matrix corresponds to a sample, and is updated after each SNP in that sample is processed, using the formula: $\boldsymbol{\theta}_{m,k} = \boldsymbol{C}_{k,m,*,*}$. Finally, the (global) $\boldsymbol{\phi}$ matrix is updated after all the samples have been processed, using the formula: $\boldsymbol{\phi}_{k,n,v} = \boldsymbol{C}_{k,*,n,v}$. Note that the $\boldsymbol{\theta}$ and $\boldsymbol{\phi}$ formula above correspond to unnormalized distributions, and can be normalized as shown in Algorithm 1 in Suppl Information. The time complexity is determined by considering the fact that the multinomial sampling is implemented using conditional binomial method with time complexity $O(K)$ (Davis 1993) in the GSL library.

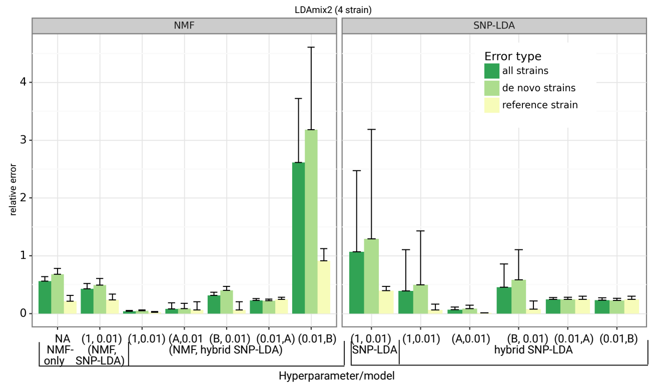

Fig. S3. Performance of different SNP-LDA model variants on LDAmix2 dataset: Model variants are indicated in a pairwise tuple format (see Suppl Section 1.3.2). Each dataset has been generated 10 times (i.e., in 10 runs using different random seeds). The x-axis indicates the different methods tested (with their hyperparameter/model combinations; see Suppl Section 1.6.2), and the y-axis indicates the relative error between the actual and predicted strain proportions averaged across the 10 runs (with error bar overall length being the standard deviation across the 10 runs). The error type indicates the error in determining all the strains, solely reference strains, and only the *de novo* strains in the samples within each dataset.

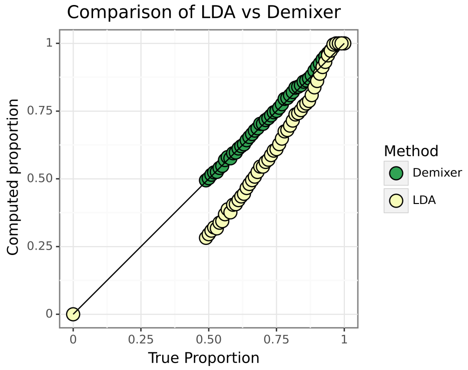

Fig. S4. Comparison of traditional text LDA with Demixer: The proportion of one of the strains estimated by traditional text LDA vs. Demixer on a 2-strain dataset (see Suppl Section 1.6.1) is compared with its true proportion.

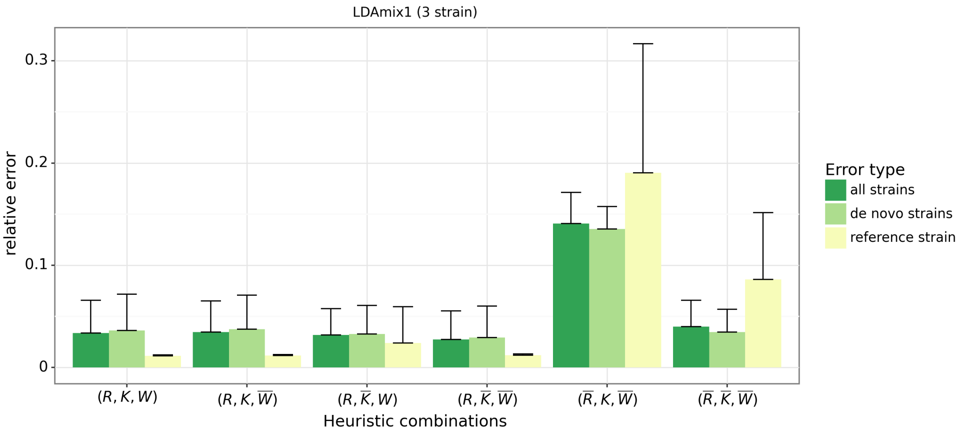

Fig. S5. Performance of the different heuristic combinations on LDAmix1 dataset: Each dataset has been generated 10 times (i.e., in 10 runs using different random seeds). The x-axis indicates the different heuristic combinations and the y-axis indicates the relative error between the actual and predicted strain proportions averaged across the 10 runs (with error bar overall length being the standard deviation across the 10 runs). The error type indicates the error in determining all the strains, solely reference strains, and only the *de novo* strains in the samples within each dataset. Here, K is set to 10 (a high value) for the combinations involving $\overline{K}$ and 3 (based on $K' + 2$ heuristic) for the combinations involving K.

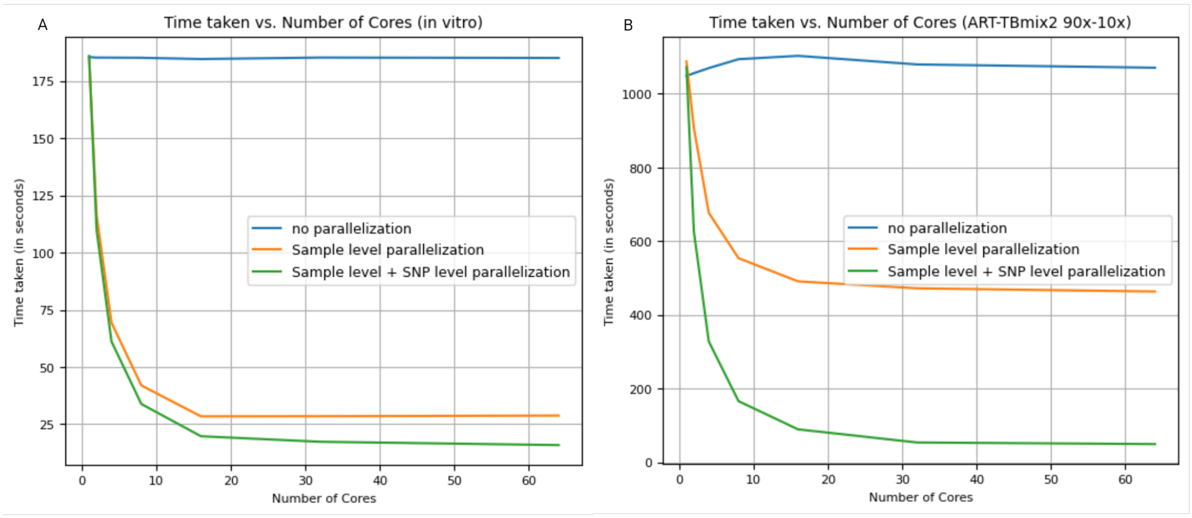

Fig. S6. Speedup from parallelization heuristic on small and medium-sized datasets: A) Time taken to run Demixer on the *in vitro* dataset with varying number of processor cores. B) Speedup plot for ART-TBmix2 (90x-10x) dataset. Sample-level parallelization corresponds to updation of z values for each individual sample, while SNP-level parallelization involves updation of the global copy of ϕ.

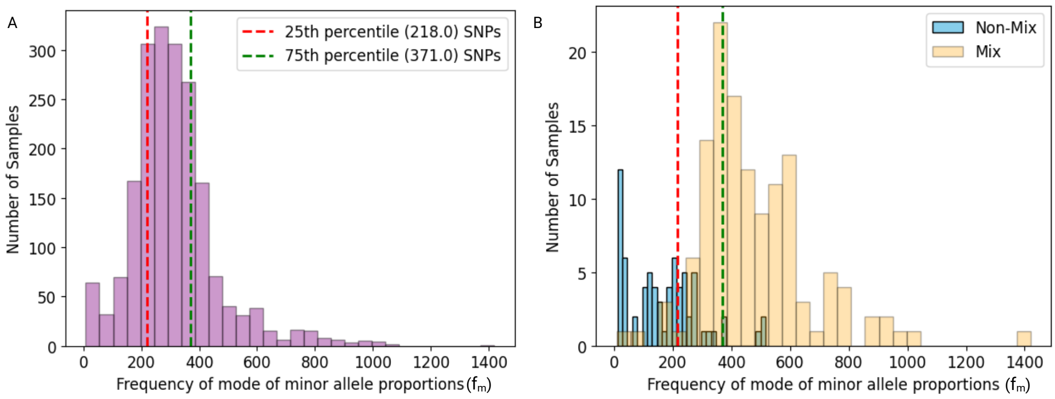

Fig. S7. Distribution of f_m values of Malawi samples: The frequency of mode of minor allele proportions, denoted f_m and expressed in terms of the number of SNPs, is used to assess the quality of mixed infection calls. A) depicts the 25th and 75th percentiles of f_m values across all samples B) shows the histogram of f_m of those samples called as mixed infection by Demixer but non-mixed by MixInfect (labelled as "Mix" and colored orange), and a second histogram corresponding to samples called as non-mixed by Demixer but mixed by MixInfect (labelled as "Non-mix" and colored blue); see also Figure 5C in main text.

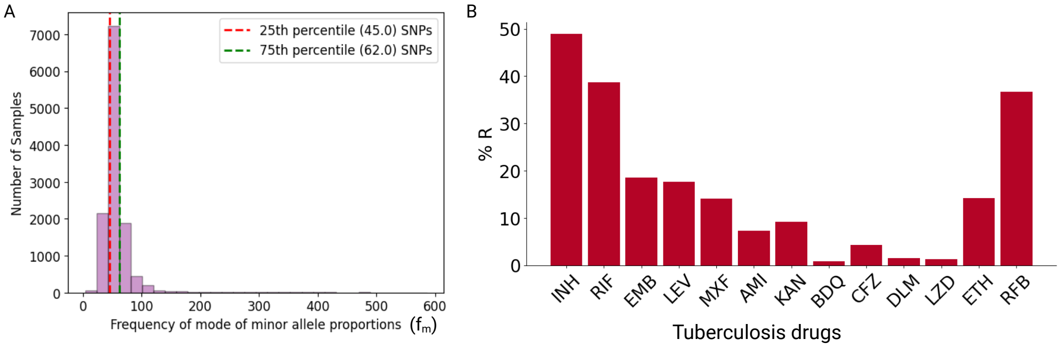

Fig. S8. Analysis of CRyPTIC samples: A) depicts the 25th and 75th percentiles of f_m across all samples. The unit of f_m is same as in previous figure. B) shows the percentage of mixed infection samples resistant to the 13 TB drugs isoniazid (INH), rifampicin (RIF), ethambutol (EMB), levofloxacin (LEV), moxifloxacin (MXF), amikacin (AMI), kanamycin (KAN), bedaquiline (BDQ), clofazimine (CFZ), delamanid (DLM), linezolid (LZD), ethionamide (ETH) and rifabutin (RFB). The figure B is generated using the opensource code from (CRyPTIC 2022a).

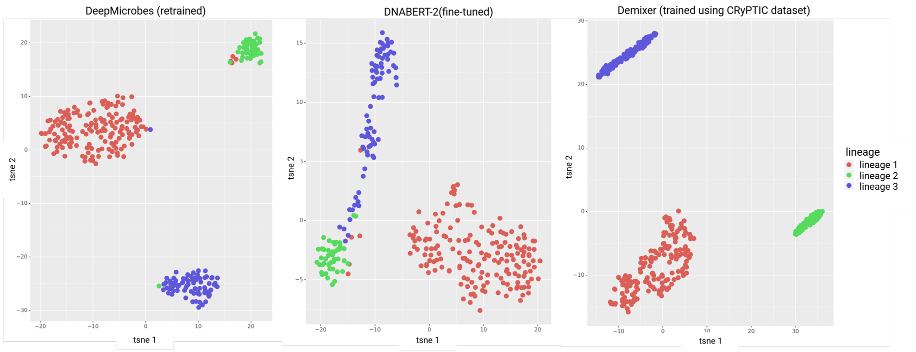

Fig. S9. tSNE plot of embeddings: The embeddings generated by DeepMicrobes, DNABERT-2, and Demixer for 300 newly simulated reads containing lineage-defining mutations (from TB lineages 1, 2 and 3) are visualized here.

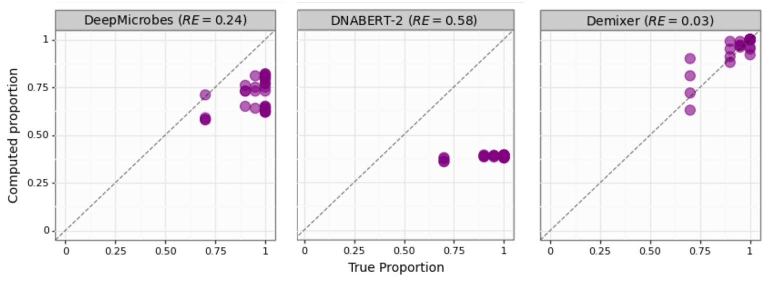

Fig. S10. Demixer vs. embedding-based methods on *in vitro* dataset: Ground-truth majority strain proportions is plotted against the proportions predicted by DeepMicrobes, DNABERT-2, and Demixer on the *in vitro* dataset. Since three lineages alone were used in training, the plots show only samples whose majority strain belongs to one of these three lineages.

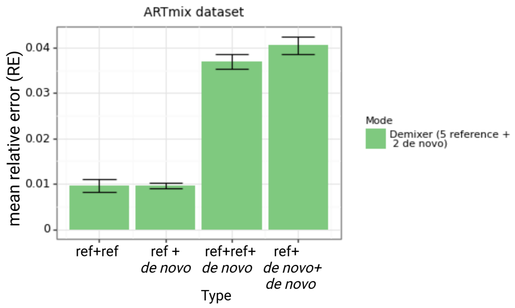

Fig. S11. Demixer in *de novo* mode: Demixer is ran on ARTmix dataset in *de novo* mode (see Section 3.3 in main text) with $\boldsymbol{wt}_{n,v}$ set to 1. The mean relative error is lower when compared to running Demixer with default weight 3 (refer Figure 4C in main text).

5. Supplementary Files

Supplementary data/result files listed below are available at this link:
https://drive.google.com/drive/folders/1P_OX_MbZ6QFN9Amyl2eGMBr1ySY6yNWu?usp=drive_link.
Suppl File D1: The actual lineages of strains mixed and the lineages of the strains determined by Demixer and QuantTB for each sample in the *in vitro* dataset.
Suppl File D2: The strains, proportions, and confidence values inferred by Demixer for each sample in the Malawi dataset.
Suppl File D3: The strains and proportions estimated by Demixer for each sample in the CRyPTIC dataset. The susceptibility of samples to all 13 drugs provided as metadata in the CRyPTIC dataset is also included here for convenience.
Suppl File D4: The strains and proportions estimated by Demixer (trained using CRyPTIC dataset) for 10 samples in the Malawi dataset.
